# Supplementary material for: Maternal Obesity Programs the Premature Aging of Rat Offspring Liver Mitochondrial Electron Transport Chain Genes in a Sex-Dependent Manner
Source: Biology (Basel). 2023 Aug 24;12(9):1166. doi: 10.3390/biology12091166 (PMC10526092; doi:10.3390/biology12091166)
Supplement: Supplementary file 1 [file biology-12-01166-s001.zip › biology-2534206-supplementary.pdf]

Supplementary Material

Table S1. Male DEG in common from the comparisons MO-110PND vs C-110PND and C-650PND vs C-110PND in males. Log2Fold change regulation and their statistical significance are shown by P-value.

		MO110 vs C110			650 vs c110		
Gene ID	Gene	Fold Regulation	P-value (t-test)	Direction	Fold Regulation	P-value (t-test)	Direction
NM_001106795	Aaas	1.36	4.64E-02	DOWN	1.50	5.06E-03	DOWN
NM_020538	Aadac	1.43	4.24E-03	DOWN	3.11	1.19E-02	DOWN
NM_001106920	Aamp	1.32	4.53E-02	DOWN	1.63	3.01E-02	DOWN
NM_001106891	Aars2	1.29	3.73E-02	DOWN	1.37	4.29E-02	DOWN
NM_031003	Abat	1.73	9.82E-04	DOWN	2.73	2.41E-02	DOWN
NM_012690	Abcb4	1.74	2.86E-02	DOWN	2.88	1.47E-02	DOWN
NM_001108201	Abcc10	1.50	3.45E-03	DOWN	1.71	1.51E-02	DOWN
NM_031013	Abcc6	1.60	1.48E-03	DOWN	2.93	3.12E-02	DOWN
NM_001013100	Abcd4	1.38	1.56E-02	DOWN	1.53	7.03E-03	DOWN
NM_001009670	Abhd14a	1.39	2.47E-02	DOWN	1.69	2.26E-02	DOWN
NM_001007664	Abhd14b	1.36	4.54E-02	DOWN	2.44	2.60E-02	DOWN
NM_016986	Acadm	1.32	2.30E-02	DOWN	1.91	4.39E-03	DOWN
NM_001267534	Accs	1.41	1.53E-02	DOWN	1.74	2.72E-02	DOWN
NM_001106111	Acot13	1.54	2.60E-02	DOWN	1.60	2.46E-02	DOWN
NM_001079709	Acot5	1.39	1.56E-02	UP	2.81	5.05E-03	DOWN
NM_016988	Acp2	1.47	1.04E-02	DOWN	2.66	9.39E-03	DOWN
NM_001031645	Acp6	1.33	2.50E-02	DOWN	1.85	9.09E-03	DOWN
NM_031144	Actb	1.54	1.71E-02	DOWN	1.59	4.24E-02	DOWN
NM_001009602	Actr10	1.27	3.38E-02	DOWN	1.53	2.66E-02	DOWN
NM_139090	Acvr1c	1.22	3.49E-02	DOWN	1.37	8.08E-03	DOWN
NM_001009603	Acy3	1.75	1.38E-02	DOWN	2.24	3.91E-02	DOWN
NM_001047101	Adamts7	2.42	1.42E-03	DOWN	4.01	3.12E-04	DOWN
NM_001108985	Adck1	1.39	1.95E-02	DOWN	1.44	7.43E-03	DOWN
NM_001107855	Adck2	1.37	2.03E-02	DOWN	1.48	2.88E-02	DOWN
NM_001126120	Adh5	1.26	3.15E-02	DOWN	1.92	1.08E-02	DOWN
NM_199097	Adi1	1.32	4.63E-02	DOWN	2.14	1.74E-02	DOWN
NM_001014144	Adtrp	1.47	4.56E-02	DOWN	4.24	3.36E-03	DOWN
NM_001111366	Afmid	1.48	1.41E-02	UP	2.72	2.35E-02	DOWN
NM_001048185	Agmat	1.61	7.55E-03	DOWN	2.63	4.76E-02	DOWN
NM_001107821	Agpat2	1.38	3.44E-02	DOWN	2.43	5.47E-03	DOWN

NM_030985	Agtr1a	1.32	3.91E-02	DOWN	2.25	4.45E-02	DOWN
NM_001276706	Agxt	2.40	2.40E-03	DOWN	2.98	2.14E-03	DOWN
NM_001271270	Ahctf1	1.59	4.43E-03	UP	1.74	1.90E-02	DOWN
NM_013149	Ahr	2.28	1.65E-02	DOWN	2.58	1.02E-02	DOWN
NM_031356	Aifm1	1.29	1.47E-02	DOWN	1.99	5.81E-03	DOWN
NM_001108497	Akip1	1.33	2.25E-02	DOWN	1.40	2.00E-02	DOWN
NM_031000	Akr1a1	1.33	3.32E-02	DOWN	1.59	1.73E-02	DOWN
NM_001135744	Akr1c12l1	1.53	9.78E-03	DOWN	2.19	5.59E-04	DOWN
NM_001014240	Akr1c13	1.61	5.82E-03	DOWN	3.09	2.51E-02	DOWN
NM_001013057	Akr1c2	2.13	1.97E-03	DOWN	2.50	4.41E-02	DOWN
NM_138510	Akr1c3	1.86	3.69E-03	DOWN	2.58	2.17E-02	DOWN
NM_134407	Akr7a2	1.35	3.51E-02	DOWN	2.46	3.75E-03	DOWN
NM_012899	Alad	1.79	5.32E-03	DOWN	2.33	2.93E-02	DOWN
NM_001033706	Aldh16a1	1.38	4.57E-02	DOWN	1.76	2.38E-02	DOWN
NM_022547	Aldh1l1	1.46	1.41E-02	DOWN	1.99	2.69E-02	DOWN
NM_032416	Aldh2	1.51	1.66E-02	DOWN	2.15	1.13E-02	DOWN
NM_022851	Aldh5a1	1.77	1.05E-04	DOWN	1.85	1.04E-02	DOWN
NM_031057	Aldh6a1	1.28	1.70E-02	DOWN	2.16	2.88E-02	DOWN
NM_022273	Aldh9a1	1.47	7.47E-04	DOWN	2.29	3.32E-02	DOWN
NM_001013951	Alg13	1.32	1.21E-02	DOWN	1.97	4.23E-03	DOWN
NM_001014176	Alg14	1.51	2.66E-02	DOWN	2.07	1.09E-02	DOWN
NM_001033709	Alg6	1.42	9.55E-04	DOWN	1.80	5.96E-04	DOWN
NM_001126273	Alkbh2	1.72	5.79E-03	DOWN	1.66	2.28E-02	DOWN
NM_001106604	Alms1	1.29	4.87E-02	DOWN	1.65	2.10E-03	DOWN
NM_001014101	Als2cr12	1.34	1.32E-02	DOWN	1.65	7.56E-03	DOWN
NM_001134341	Ambra1	1.38	2.01E-02	DOWN	1.45	3.25E-02	DOWN
NM_031011	Amd1	1.58	9.49E-03	DOWN	1.55	4.67E-02	DOWN
NM_001191781	Amdhd1	1.96	2.96E-02	DOWN	2.75	4.72E-02	DOWN
NM_001101681	Ampd2	1.45	8.42E-03	DOWN	1.96	3.05E-02	DOWN
NM_001014004	Amt	1.40	4.88E-03	DOWN	2.06	3.58E-02	DOWN
NM_001173983	Anapc13	1.38	2.14E-02	DOWN	1.74	1.52E-02	DOWN
NM_001108717	Angel1	1.45	2.64E-03	DOWN	1.50	3.49E-02	DOWN
NM_001033698	Ankrd16	1.37	1.74E-02	DOWN	1.59	1.18E-02	DOWN
NM_001013948	Ankrd46	1.65	3.59E-03	DOWN	1.71	3.20E-02	DOWN
NM_001025285	Ankrd54	1.55	2.82E-03	DOWN	1.54	1.19E-02	DOWN
NM_001134969	Ankrd6	1.13	2.10E-02	DOWN	1.19	3.26E-02	UP
NM_001127650	Anks4b	1.40	3.07E-02	DOWN	2.84	3.08E-02	DOWN
NM_012903	Anp32a	1.59	7.62E-04	DOWN	1.70	1.32E-02	DOWN
NM_001044239	Ap1m1	1.23	4.07E-02	DOWN	1.56	2.31E-02	DOWN
NM_031008	Ap2a2	1.31	3.10E-02	DOWN	1.41	3.02E-02	DOWN
NM_133593	Ap3m1	1.32	4.66E-02	DOWN	1.48	3.69E-02	DOWN

NM_001115039	Ap3s2	1.48	9.05E-03	DOWN	1.59	1.15E-02	DOWN
NM_031781	Apba3	1.35	3.10E-02	DOWN	1.58	1.60E-02	DOWN
NM_001106492	Apip	1.31	2.86E-02	DOWN	1.42	1.35E-02	DOWN
NM_001173382	Aplf	1.52	2.31E-02	DOWN	1.57	2.00E-02	DOWN
NM_001034003	Apmap	1.43	1.69E-02	DOWN	2.41	7.26E-03	DOWN
NM_001106440	Apoa1bp	1.34	3.26E-02	DOWN	1.69	8.69E-03	DOWN
NM_012824	Apoc1	1.45	1.42E-02	DOWN	3.61	8.07E-03	DOWN
NM_001014105	Apool	1.41	5.62E-03	DOWN	1.57	6.64E-03	DOWN
NM_148889	Aptx	1.35	4.54E-02	DOWN	1.55	3.36E-02	DOWN
NM_173105	Aqp11	1.26	4.94E-02	DOWN	2.12	1.86E-02	DOWN
NM_022532	Araf	1.28	1.06E-02	DOWN	1.71	2.32E-02	DOWN
NM_024150	Arf2	1.31	9.54E-03	DOWN	1.65	3.82E-03	DOWN
NM_024151	Arf4	1.26	4.68E-02	DOWN	1.77	2.67E-02	DOWN
NM_024149	Arf5	1.45	6.61E-03	DOWN	1.73	1.60E-02	DOWN
NM_145090	Arfgap1	1.39	8.79E-03	DOWN	1.38	3.75E-02	DOWN
NM_001033707	Arfgap2	1.52	2.98E-03	DOWN	1.42	4.11E-02	DOWN
NM_001107357	Arhgap12	1.50	1.73E-02	DOWN	1.52	3.08E-02	DOWN
NM_001012032	Arhgap24	1.67	1.15E-02	DOWN	2.08	4.37E-03	DOWN
NM_001271132	Arhgap35	1.29	4.12E-02	DOWN	1.81	1.40E-02	DOWN
NM_021694	Arhgef1	1.33	1.55E-02	DOWN	1.53	3.48E-02	DOWN
NM_023982	Arhgef11	1.33	3.16E-02	DOWN	1.66	8.40E-03	DOWN
NM_001107115	Arhgef18	1.32	3.78E-02	DOWN	1.78	9.17E-03	DOWN
NM_001108692	Arhgef19	2.43	2.02E-05	DOWN	2.31	4.70E-02	DOWN
NM_022700	Arl3	1.39	2.19E-02	DOWN	1.74	9.53E-03	DOWN
NM_001106919	Arpc2	1.30	4.15E-02	DOWN	1.78	6.81E-03	DOWN
NM_001106615	Arpc4	1.38	1.22E-02	DOWN	1.66	5.92E-03	DOWN
NM_001025717	Arpc5	1.22	4.97E-02	DOWN	1.75	2.99E-02	DOWN
NM_001107530	Arpin	1.43	1.49E-02	DOWN	1.50	2.83E-02	DOWN
NM_001034933	Arsa	1.74	1.35E-03	DOWN	1.76	1.50E-02	DOWN
NM_001108864	Asb3	1.39	8.97E-03	DOWN	1.49	1.35E-02	DOWN
NM_001009643	Aspdh	1.42	5.55E-03	DOWN	2.82	2.36E-02	DOWN
NM_001040156	Aste1	1.21	1.50E-02	DOWN	1.34	1.40E-03	DOWN
NM_172336	Atf5	1.96	8.18E-03	DOWN	3.09	1.09E-03	DOWN
NM_001002809	Atf6b	1.42	2.03E-02	DOWN	1.51	2.95E-02	DOWN
NM_134394	Atg3	1.36	1.31E-02	DOWN	1.85	2.68E-03	DOWN
NM_001126298	Atg4al1	1.35	7.08E-03	DOWN	1.86	1.77E-03	DOWN
NM_053359	Atox1	1.52	2.97E-02	DOWN	2.50	1.33E-02	DOWN
NM_001107324	Atp11a	1.56	3.62E-03	DOWN	1.61	5.76E-03	DOWN
NM_001109369	Atp13a3	1.23	4.74E-02	DOWN	1.25	2.71E-02	DOWN
NM_053825	Atp5c1	1.33	7.33E-03	DOWN	1.56	2.37E-02	DOWN
NM_080481	Atp5i	1.46	2.73E-02	DOWN	1.68	2.15E-02	DOWN

NM_138883	Atp5o	1.34	3.65E-02	DOWN	1.67	1.89E-02	DOWN
NM_001007749	Atp5s	1.34	8.02E-03	DOWN	1.76	1.34E-02	DOWN
NM_053884	Atp6v1f	1.30	4.82E-02	DOWN	1.58	2.08E-02	DOWN
NM_001106130	Atp9b	1.40	4.60E-03	DOWN	1.47	1.70E-02	DOWN
NM_001107959	Atpaf1	1.39	1.44E-03	DOWN	1.44	1.20E-02	DOWN
NM_001127526	Atraid	1.43	3.07E-03	DOWN	1.90	2.27E-02	DOWN
NM_001136261	Atxn7l3b	1.22	3.99E-02	DOWN	1.87	1.87E-02	DOWN
NM_001079899	Aup1	1.32	4.77E-02	DOWN	1.99	4.96E-03	DOWN
NM_001004237	Aurkaip1	1.33	3.55E-02	DOWN	1.68	1.53E-02	DOWN
NM_024355	Axin2	1.72	1.30E-02	DOWN	1.83	1.43E-02	DOWN
NM_001128184	B3gat3	1.35	3.53E-02	DOWN	1.63	2.55E-02	DOWN
NM_001106324	B3gnt1	1.24	1.42E-02	DOWN	1.59	9.98E-03	DOWN
NM_022860	B4galnt1	1.99	2.34E-02	DOWN	2.32	4.25E-02	DOWN
NM_022698	Bad	1.44	1.21E-02	DOWN	1.78	2.05E-02	DOWN
NM_139082	Bambi	1.70	4.41E-03	DOWN	1.48	4.86E-02	DOWN
NM_001107158	Baz2a	1.38	3.83E-02	DOWN	1.60	1.07E-02	DOWN
NM_022629	Bbox1	1.34	1.89E-02	DOWN	2.69	2.20E-02	DOWN
NM_001109286	Bbs10	1.83	9.12E-03	DOWN	1.93	1.93E-02	DOWN
NM_001106826	Bbs4	1.53	3.19E-02	DOWN	1.70	1.09E-02	DOWN
NM_001006980	Bcap29	1.33	6.09E-03	DOWN	1.66	4.22E-03	DOWN
NM_001107722	Bcar3	1.86	2.49E-02	DOWN	1.72	3.47E-02	DOWN
NM_001106458	Bcas2	1.34	6.37E-03	DOWN	1.79	3.01E-03	DOWN
NM_012782	Bckdha	1.40	1.04E-03	DOWN	1.52	4.80E-02	DOWN
NM_019267	Bckdhb	1.42	1.31E-02	DOWN	1.94	4.12E-03	DOWN
NM_001127712	Bco2	1.52	2.31E-03	DOWN	3.15	1.56E-02	DOWN
NM_053995	Bdh1	1.60	4.95E-03	DOWN	3.11	1.32E-02	DOWN
NM_019251	Bet1	1.34	6.05E-03	DOWN	1.86	1.47E-02	DOWN
NM_207611	Bhlhb9	1.43	2.44E-02	DOWN	1.75	1.72E-02	DOWN
NM_030850	Bhmt	2.90	5.50E-04	DOWN	3.31	3.94E-02	DOWN
NM_022684	Bid	1.43	3.49E-02	DOWN	1.60	1.63E-02	DOWN
NM_001012223	Bin2	1.72	2.39E-02	DOWN	1.78	3.41E-02	DOWN
NM_001107347	Bloc1s5	1.48	1.26E-03	DOWN	1.58	8.58E-03	DOWN
NM_053850	Blvra	1.50	1.07E-02	DOWN	1.71	2.46E-02	DOWN
NM_139258	Bmf	1.95	7.02E-05	DOWN	2.69	5.53E-03	DOWN
NM_031323	Bmp1	1.77	1.39E-04	DOWN	2.12	6.23E-03	DOWN
NM_001037206	Bphl	1.40	7.45E-03	DOWN	2.69	6.35E-03	DOWN
NM_001108440	Brd7	1.31	1.56E-02	DOWN	1.46	2.52E-02	DOWN
NM_199270	Bre	1.34	3.36E-02	DOWN	1.63	2.45E-02	DOWN
NM_001012171	Bscl2	1.46	1.27E-02	DOWN	1.92	8.63E-03	DOWN
NM_022261	Bspry	1.54	1.70E-02	DOWN	1.39	2.49E-02	DOWN
NM_001077683	Btbd6	1.36	1.88E-02	DOWN	1.51	2.23E-02	DOWN

NM_001047906	Bub3	1.35	1.35E-02	DOWN	1.66	1.03E-02	DOWN
NM_199391	C2cd2	1.53	8.58E-05	DOWN	1.82	1.94E-03	DOWN
NM_001108926	Cabp4	1.56	5.34E-03	DOWN	1.58	1.30E-02	DOWN
NM_001143893	Cabyr	1.28	4.62E-02	DOWN	1.38	7.63E-03	DOWN
NM_017298	Cacna1d	1.77	5.48E-03	DOWN	1.99	2.98E-02	DOWN
NM_017326	Calm2	1.44	8.20E-03	DOWN	1.42	4.49E-02	DOWN
NM_001105801	Camta2	1.33	1.82E-02	DOWN	1.49	2.77E-02	DOWN
NM_031673	Capn10	1.48	1.45E-03	DOWN	1.50	2.65E-02	DOWN
NM_001030037	Capn7	1.27	3.73E-02	DOWN	1.55	1.55E-02	DOWN
NM_017118	Capns1	1.35	2.77E-02	DOWN	1.59	1.25E-02	DOWN
NM_001108990	Caps2	1.39	2.78E-02	DOWN	1.61	1.22E-02	DOWN
NM_001009180	Capza2	1.22	3.73E-02	DOWN	1.58	2.36E-02	DOWN
NM_001106165	Car7	1.48	2.40E-02	DOWN	1.52	2.78E-02	DOWN
NM_001177684	Cars2	1.42	6.44E-03	DOWN	1.74	6.83E-03	DOWN
NM_012922	Casp3	1.42	2.79E-02	DOWN	2.29	2.52E-02	DOWN
NM_022260	Casp7	1.65	6.95E-04	DOWN	1.60	3.98E-02	DOWN
NM_001033715	Cast	1.27	9.31E-03	DOWN	1.69	1.47E-02	DOWN
NM_012520	Cat	1.57	9.61E-04	DOWN	2.78	2.08E-02	DOWN
NM_182672	Cbr4	1.34	1.27E-02	DOWN	1.80	2.52E-03	DOWN
NM_012522	Cbs	1.58	3.01E-03	DOWN	2.74	3.52E-02	DOWN
NM_001034078	Cbx8	1.35	2.54E-02	DOWN	1.72	1.72E-02	DOWN
NM_001013869	Cc2d1a	1.46	1.04E-03	DOWN	1.71	2.04E-03	DOWN
NM_001024866	Ccdc104	1.24	3.38E-02	DOWN	1.96	1.06E-03	DOWN
NM_001014067	Ccdc17	1.48	1.19E-02	DOWN	2.16	2.21E-03	DOWN
NM_001134688	Ccdc40	1.07	3.66E-02	UP	1.13	4.52E-02	UP
NM_001014098	Ccdc51	1.41	2.30E-02	DOWN	1.37	4.88E-02	DOWN
NM_001105875	Ccdc58	1.33	3.17E-02	DOWN	2.25	1.14E-03	DOWN
NM_001108660	Ccl27	1.46	1.60E-02	DOWN	1.50	2.43E-02	DOWN
NM_001037794	Cct8l1	1.12	1.28E-02	DOWN	1.14	1.94E-03	DOWN
NM_031812	Cd164	1.59	4.54E-04	DOWN	1.91	4.48E-02	DOWN
NM_001013237	Cd19	1.46	6.54E-03	DOWN	1.68	4.65E-04	DOWN
NM_170789	Cd247	1.74	4.16E-02	DOWN	1.77	1.22E-02	DOWN
NM_013169	Cd3d	1.23	1.82E-02	DOWN	1.22	1.83E-02	DOWN
NM_012925	Cd59	1.45	6.15E-03	DOWN	1.64	1.47E-02	DOWN
NM_175577	Cd6	1.35	3.63E-02	DOWN	1.37	1.65E-02	DOWN
NM_053877	Cdc123	1.27	3.73E-02	DOWN	1.80	4.85E-03	DOWN
NM_001024744	Cdc16	1.25	1.55E-02	DOWN	1.52	1.93E-02	DOWN
NM_001100659	Cdc23	1.36	2.58E-02	DOWN	1.48	1.46E-02	DOWN
NM_001013240	Cdc26	1.31	6.06E-03	DOWN	1.89	1.83E-02	DOWN
NM_138899	Cdipt	1.36	1.75E-02	DOWN	1.76	6.94E-03	DOWN
NM_001109937	Cdk10	2.09	2.58E-02	DOWN	1.62	3.71E-02	DOWN

NM_001025752	Cdk20	1.24	4.21E-02	DOWN	1.50	2.66E-02	DOWN
NM_001113751	Cdk2ap1	1.32	2.63E-02	DOWN	1.62	3.13E-02	DOWN
NM_001109498	Cdk2ap2	1.70	1.32E-02	DOWN	1.86	4.62E-02	DOWN
NM_053593	Cdk4	1.41	1.90E-02	DOWN	1.43	4.60E-02	DOWN
NM_080885	Cdk5	1.23	2.94E-02	DOWN	1.42	3.11E-02	DOWN
NM_001033862	Ceacam1	1.92	5.09E-03	DOWN	2.70	1.35E-02	DOWN
NM_001004098	Cenpc	1.41	9.28E-03	DOWN	1.56	3.44E-02	DOWN
NM_001107265	Cenpj	1.33	4.85E-02	DOWN	1.73	2.49E-02	DOWN
NM_001244761	Cenpl	1.98	2.59E-03	DOWN	1.61	9.93E-03	DOWN
NM_001108124	Cep57	1.30	2.34E-02	DOWN	1.43	3.15E-02	DOWN
NM_001013862	Cep95	1.44	1.84E-03	DOWN	1.54	1.57E-02	DOWN
NM_001033700	Cers2	1.39	3.26E-02	DOWN	2.62	1.27E-02	DOWN
NM_199378	Cfdp1	1.45	8.19E-03	DOWN	1.56	6.28E-03	DOWN
NM_001108369	Chchd1	1.41	4.64E-02	DOWN	1.62	3.37E-02	DOWN
NM_001107797	Chd6	1.26	2.78E-02	DOWN	1.38	3.95E-02	DOWN
NM_001108906	Chmp2a	1.38	4.72E-02	DOWN	1.59	2.50E-02	DOWN
NM_001025410	Chmp5	1.32	2.46E-02	DOWN	1.80	1.14E-02	DOWN
NM_001108872	Chmp7	1.35	1.07E-02	DOWN	1.60	2.07E-02	DOWN
NM_001106574	Chpf2	1.40	2.85E-02	DOWN	1.76	1.44E-02	DOWN
NM_057134	Chrd	1.64	3.05E-03	DOWN	1.84	3.95E-02	DOWN
NM_001108869	Cideb	1.28	1.43E-02	DOWN	2.77	2.99E-02	DOWN
NM_001270803	Ciita	1.88	1.25E-02	DOWN	1.83	2.06E-02	DOWN
NM_001105835	Cisd3	1.51	2.55E-02	DOWN	1.86	4.39E-03	DOWN
NM_031699	Cldn1	1.46	4.88E-02	DOWN	3.56	7.84E-04	DOWN
NM_001014254	Clk2	1.33	1.29E-02	DOWN	1.61	1.32E-02	DOWN
NM_019299	Cltc	1.32	3.81E-02	DOWN	1.58	3.36E-02	DOWN
NM_001014225	Cluap1	1.27	7.53E-03	DOWN	1.39	4.40E-02	DOWN
NM_001108969	Clvs1	1.20	6.06E-03	DOWN	1.55	1.14E-02	DOWN
NM_198754	Cmtm8	1.49	1.81E-03	DOWN	2.10	3.53E-02	DOWN
NM_022598	Cnbp	1.24	4.88E-02	DOWN	1.59	2.01E-02	DOWN
NM_001106173	Cnep1r1	1.47	2.67E-03	DOWN	1.39	1.09E-02	DOWN
NM_001106029	Cnih1	1.29	2.44E-02	DOWN	1.89	1.69E-03	DOWN
NM_001105981	Cnih4	1.29	2.91E-02	DOWN	1.54	7.37E-03	DOWN
NM_001106901	Cnnm3	1.48	1.01E-02	DOWN	1.64	2.31E-02	DOWN
NM_001007003	Cnot10	1.34	2.91E-02	DOWN	1.57	6.66E-03	DOWN
NM_001077585	Cnpy2	1.37	1.46E-02	DOWN	1.85	2.07E-02	DOWN
NM_001006954	Coasy	1.38	1.59E-02	DOWN	1.86	2.39E-02	DOWN
NM_001108449	Cog4	1.25	4.70E-02	DOWN	1.78	2.48E-03	DOWN
NM_032085	Col3a1	1.31	5.02E-03	DOWN	1.50	2.66E-02	DOWN
NM_001115022	Commd1	1.34	1.30E-02	DOWN	1.47	8.38E-03	DOWN
NM_001004276	Commd10	1.35	4.65E-03	DOWN	1.57	3.67E-02	DOWN

NM_001109503	Commd2	1.42	2.93E-03	DOWN	1.91	1.20E-03	DOWN
NM_198732	Commd3	1.36	3.08E-02	DOWN	1.83	5.64E-03	DOWN
NM_001108762	Commd4	1.46	1.78E-02	DOWN	1.53	4.17E-02	DOWN
NM_139108	Commd5	1.53	4.25E-02	DOWN	1.86	2.03E-02	DOWN
NM_001030029	Commd7	1.71	8.99E-04	DOWN	1.45	2.11E-02	DOWN
NM_001106076	Cope	1.26	4.24E-02	DOWN	1.55	1.87E-02	DOWN
NM_001106929	Copg2	1.48	1.60E-03	DOWN	1.48	1.10E-02	DOWN
NM_001025695	Cops5	1.34	1.58E-02	DOWN	1.68	1.27E-02	DOWN
NM_001108807	Cops7b	1.26	8.49E-03	DOWN	1.48	1.90E-02	DOWN
NM_001013227	Cops8	1.33	3.15E-02	DOWN	1.79	4.85E-03	DOWN
NM_001108294	Copz2	1.39	1.86E-02	DOWN	1.50	1.75E-02	DOWN
NM_001031662	Coq4	1.49	2.27E-03	DOWN	1.40	3.63E-02	DOWN
NM_001039022	Coq5	1.61	6.70E-03	DOWN	1.72	8.66E-03	DOWN
NM_001033699	Cox15	1.47	1.54E-02	DOWN	1.56	4.90E-02	DOWN
NM_001163153	Cox16	1.26	5.00E-02	DOWN	2.16	2.65E-03	DOWN
NM_001105976	Cox20	1.36	3.24E-02	DOWN	1.63	6.14E-03	DOWN
NM_053586	Cox5b	1.34	4.65E-02	DOWN	1.71	1.57E-02	DOWN
NM_001106704	Cox7a2l	1.28	2.93E-02	DOWN	1.47	1.98E-02	DOWN
NM_001108454	Cpne7	1.34	2.92E-02	DOWN	1.54	3.77E-02	DOWN
NM_001037095	Cpox	1.36	4.28E-02	DOWN	1.75	1.48E-02	DOWN
NM_031640	Cpq	1.29	4.42E-02	DOWN	2.50	2.00E-02	DOWN
NM_001130571	Cpsf1	1.25	4.18E-02	DOWN	1.69	5.41E-03	DOWN
NM_053670	Crcp	1.46	4.18E-02	DOWN	1.93	1.59E-02	DOWN
NM_022501	Crip2	1.35	8.17E-03	DOWN	1.70	3.39E-02	DOWN
NM_031987	Crot	1.98	1.53E-02	DOWN	2.84	1.93E-02	DOWN
NM_001033895	Crtc2	1.32	4.38E-02	DOWN	1.53	4.28E-02	DOWN
NM_133405	Cry2	1.40	2.98E-02	DOWN	1.87	9.05E-03	DOWN
NM_031021	Csnk2b	1.34	1.16E-02	DOWN	1.55	2.05E-02	DOWN
NM_031023	Ctbs	1.19	4.17E-02	DOWN	2.20	3.59E-02	DOWN
NM_001128079	Ctdsp1	1.37	1.81E-02	DOWN	1.61	2.18E-02	DOWN
NM_001024870	Ctnnbl1	1.33	1.35E-02	DOWN	1.63	1.48E-02	DOWN
NM_012939	Ctsh	1.50	7.50E-03	DOWN	2.03	4.20E-02	DOWN
NM_001079886	Cuedc2	1.38	2.20E-02	DOWN	1.40	4.42E-02	DOWN
NM_001191582	Cul9	1.36	4.40E-03	DOWN	1.37	4.02E-02	DOWN
NM_001108525	Cutc	1.35	4.46E-02	DOWN	2.01	1.84E-03	DOWN
NM_001013199	Cwc27	1.34	1.74E-02	DOWN	1.49	8.71E-03	DOWN
NM_022177	Cxcl12	1.64	1.52E-02	DOWN	2.03	4.05E-02	DOWN
NM_001007753	Cyb561d2	1.45	1.39E-02	DOWN	1.59	4.96E-02	DOWN
NM_030586	Cyb5b	1.25	2.25E-02	DOWN	1.56	4.34E-02	DOWN
NM_138877	Cyb5r3	1.44	6.05E-03	DOWN	2.09	5.49E-03	DOWN
NM_133427	Cyb5r4	1.36	3.46E-02	DOWN	1.76	5.05E-03	DOWN

NM_012538	Cyp11b2	1.08	4.47E-02	DOWN	1.10	4.61E-02	DOWN
NM_012693	Cyp2a2	1.83	3.51E-04	DOWN	3.40	1.64E-02	DOWN
NM_173294	Cyp2b3	1.25	4.07E-02	DOWN	3.24	2.59E-02	DOWN
NM_012730	Cyp2d2	1.41	1.88E-02	DOWN	3.36	1.28E-02	DOWN
NM_173093	Cyp2d3	1.37	2.59E-02	DOWN	3.17	1.47E-02	DOWN
NM_138515	Cyp2d4	1.78	3.56E-02	DOWN	5.55	1.85E-04	DOWN
NM_173304	Cyp2d5	1.76	1.70E-02	DOWN	3.78	5.10E-03	DOWN
NM_001024779	Cyp2u1	2.00	2.09E-02	DOWN	2.92	7.92E-04	DOWN
NM_153312	Cyp3a2	1.88	2.49E-02	DOWN	4.25	2.10E-02	DOWN
NM_019623	Cyp4f1	1.36	1.68E-02	DOWN	3.03	2.55E-02	DOWN
NM_153318	Cyp4f6	1.40	1.78E-02	DOWN	2.17	5.58E-03	DOWN
NM_031241	Cyp8b1	3.21	5.71E-03	DOWN	2.62	4.88E-02	DOWN
NM_001106926	D2hgdh	1.43	3.82E-03	DOWN	1.97	1.95E-02	DOWN
NM_138910	Dad1	1.26	4.82E-02	DOWN	1.69	2.61E-02	DOWN
NM_001107120	Daglb	1.36	4.25E-03	DOWN	1.63	6.49E-03	DOWN
NM_001011950	Dap3	1.35	1.34E-03	DOWN	1.42	1.76E-02	DOWN
NM_001013107	Dazap2	1.38	9.41E-03	DOWN	1.91	2.62E-03	DOWN
NM_021596	Dbil5	1.13	2.73E-02	DOWN	1.29	3.55E-04	DOWN
NM_001277212	Dbnl	1.60	7.24E-03	DOWN	1.84	1.38E-02	DOWN
NM_001009686	Dcaf11	1.51	7.85E-03	DOWN	2.32	1.05E-02	DOWN
NM_001107162	Dcaf15	1.36	4.51E-02	DOWN	1.49	3.34E-02	DOWN
NM_001107057	Dcaf7	1.21	9.41E-03	DOWN	1.33	4.42E-02	DOWN
NM_001007724	Dcakd	1.63	3.61E-03	DOWN	1.61	1.42E-02	DOWN
NM_001108659	Dctn3	1.30	4.05E-02	DOWN	1.58	2.27E-02	DOWN
i	Ddc	1.77	3.29E-03	DOWN	2.31	4.92E-02	DOWN
NM_024131	Ddt	1.41	3.46E-02	DOWN	2.38	1.80E-02	DOWN
NM_001015018	Ddx17	1.32	3.09E-02	DOWN	1.46	1.46E-02	DOWN
NM_001108046	Ddx41	1.25	4.53E-02	DOWN	1.53	4.75E-02	DOWN
NM_001191747	Dennd1a	1.39	2.72E-02	DOWN	1.60	2.57E-02	DOWN
NM_001126289	Dennd1c	1.44	1.75E-02	DOWN	1.59	4.77E-02	DOWN
NM_001029916	Depdc7	1.24	1.10E-02	DOWN	2.63	3.83E-02	DOWN
NM_001014202	Derl1	1.29	2.47E-02	DOWN	1.77	1.56E-02	DOWN
NM_001109577	Derl3	1.43	2.70E-02	UP	1.71	8.87E-03	DOWN
NM_053362	Dffb	1.46	1.42E-02	DOWN	1.57	2.06E-02	DOWN
NM_001012345	Dgat2	1.67	8.54E-03	DOWN	2.17	2.34E-02	DOWN
NM_001106602	Dguok	1.28	3.36E-02	DOWN	1.54	9.96E-03	DOWN
NM_001007621	Dhrs1	1.40	3.41E-02	DOWN	1.88	4.39E-02	DOWN
NM_001037199	Dhrs3	1.39	2.40E-02	DOWN	2.26	3.57E-02	DOWN
NM_001130039	Dhx32	1.34	3.07E-02	DOWN	1.44	2.74E-02	DOWN
NM_001106185	Dhx38	1.33	1.82E-02	DOWN	1.58	1.62E-02	DOWN
NM_001008380	Dis3l	1.34	1.11E-02	DOWN	1.48	2.86E-02	DOWN

NM_199385	Dld	1.36	1.28E-03	DOWN	1.58	5.26E-03	DOWN
NM_032063	Dll1	2.36	3.06E-04	DOWN	1.62	3.61E-02	DOWN
NM_001015006	Dmap1	1.58	4.17E-03	DOWN	1.51	1.60E-02	DOWN
NM_001108385	Dmtn	1.98	4.42E-03	DOWN	1.71	4.67E-02	DOWN
NM_001130510	Dnajb7	1.45	6.18E-03	DOWN	1.52	1.30E-03	DOWN
NM_001034032	Dnajc12	1.91	1.05E-02	DOWN	2.23	4.52E-03	DOWN
NM_001106050	Dnajc15	1.27	4.17E-02	DOWN	1.63	2.68E-02	DOWN
NM_001014204	Dnajc22	1.31	3.16E-02	DOWN	2.29	4.08E-02	DOWN
NM_001191853	Dnajc24	1.24	3.61E-02	DOWN	1.67	1.54E-03	DOWN
NM_022232	Dnajc3	1.26	2.59E-02	DOWN	2.39	3.11E-02	DOWN
NM_001109024	Dnajc30	1.26	3.65E-02	DOWN	1.78	1.04E-02	DOWN
NM_053354	Dnmt1	1.44	4.75E-03	DOWN	1.41	1.98E-02	DOWN
NM_001003959	Dnmt3b	1.47	4.46E-02	DOWN	1.71	2.38E-02	DOWN
NM_001143858	Dock1	1.39	1.52E-02	DOWN	1.43	3.95E-02	DOWN
NM_001107826	Dolk	1.30	4.60E-02	DOWN	1.80	4.90E-03	DOWN
NM_199388	Dpagt1	1.26	4.09E-02	DOWN	1.49	4.09E-02	DOWN
NM_001014181	Dph6	1.34	3.11E-03	DOWN	1.38	1.64E-02	DOWN
NM_019252	Dpm2	1.33	1.61E-02	DOWN	1.71	1.90E-02	DOWN
NM_001109331	Dpm3	1.27	4.22E-02	DOWN	1.74	7.69E-03	DOWN
NM_053748	Dpp3	1.42	1.44E-02	DOWN	2.31	5.08E-03	DOWN
NM_001077668	Drap1	1.34	4.24E-02	DOWN	1.49	3.52E-02	DOWN
NM_001108594	Dtd1	1.43	4.75E-03	DOWN	1.41	3.75E-02	DOWN
NM_001108981	Dtd2	1.45	3.32E-03	DOWN	1.95	8.62E-03	DOWN
NM_001037664	Dtnbp1	1.63	7.32E-03	DOWN	1.74	2.20E-03	DOWN
NM_001013921	Dtwd1	1.42	2.84E-03	DOWN	1.36	2.39E-03	DOWN
NM_053769	Dusp1	1.61	1.57E-02	UP	2.62	6.33E-03	DOWN
NM_031820	Dvl1	1.31	4.34E-02	DOWN	1.68	1.15E-02	DOWN
NM_001270625	Dync1i2	1.33	2.83E-02	DOWN	2.24	2.64E-03	DOWN
NM_001108100	Dyrk2	1.33	2.86E-02	DOWN	1.93	4.04E-03	DOWN
NM_001134987	Eapp	1.33	2.39E-02	DOWN	1.92	2.62E-03	DOWN
NM_001108381	Ebpl	1.34	1.39E-02	DOWN	2.05	1.87E-02	DOWN
NM_001106675	Echdc2	1.33	4.11E-02	DOWN	2.49	1.28E-02	DOWN
NM_001101010	Echdc3	1.32	3.10E-03	DOWN	2.48	4.99E-02	DOWN
NM_078623	Echs1	1.42	3.57E-04	DOWN	1.89	1.60E-02	DOWN
NM_001006986	Ecsit	1.40	6.54E-03	DOWN	1.57	3.40E-02	DOWN
NM_001106557	Edf1	1.45	1.90E-02	DOWN	1.62	2.16E-02	DOWN
NM_001109249	Eefsec	1.30	4.10E-02	DOWN	1.48	2.96E-02	DOWN
NM_012842	Egf	1.78	6.99E-03	DOWN	2.39	6.10E-03	DOWN
NM_001004083	Egln2	1.31	1.04E-02	DOWN	1.70	4.74E-03	DOWN
NM_212463	Ehmt2	1.51	3.43E-03	DOWN	1.62	3.10E-02	DOWN
NM_001025660	Ei24	1.56	8.82E-03	DOWN	2.35	1.06E-02	DOWN

NM_001100542	Eif2s3x	1.28	2.12E-02	DOWN	1.50	1.95E-02	DOWN
NM_001134955	Elmo2	1.42	3.68E-03	DOWN	1.42	2.13E-02	DOWN
NM_001109506	Elmod2	1.51	7.34E-03	DOWN	2.03	3.68E-03	DOWN
NM_001126098	Elof1	1.37	1.63E-02	DOWN	1.64	2.71E-02	DOWN
NM_134382	Elovl5	1.99	3.86E-02	DOWN	3.01	1.57E-02	DOWN
NM_001108782	Elp6	1.40	3.05E-02	DOWN	1.57	9.76E-03	DOWN
NM_001010968	Eng	1.64	8.48E-03	DOWN	2.07	3.04E-02	DOWN
NM_012554	Eno1	1.28	4.16E-02	DOWN	1.52	3.29E-02	DOWN
NM_001100729	Enthd2	1.42	7.00E-03	DOWN	1.57	3.44E-02	DOWN
NM_001033565	Entpd8	1.71	1.45E-03	DOWN	2.91	2.11E-02	DOWN
NM_001107858	Epha1	1.37	2.14E-02	DOWN	2.08	3.63E-02	DOWN
NM_001271384	Epm2aip1	1.31	3.65E-02	DOWN	1.60	1.77E-02	DOWN
NM_001108508	Eps8l2	1.41	6.36E-03	DOWN	1.54	2.44E-02	DOWN
NM_001172809	Ercc2	1.29	2.13E-02	DOWN	1.61	2.08E-02	DOWN
NM_001031644	Ercc3	1.39	8.04E-03	DOWN	1.60	1.62E-02	DOWN
NM_184050	Ermp1	1.19	6.28E-03	DOWN	1.49	4.72E-03	DOWN
NM_053961	Erp29	1.41	1.13E-02	DOWN	1.67	3.39E-02	DOWN
NM_001008317	Erp44	1.24	2.64E-02	DOWN	2.07	7.11E-03	DOWN
NM_198742	Etfdh	1.37	2.69E-02	DOWN	2.12	6.13E-04	DOWN
NM_001109243	Eva1a	1.70	2.92E-02	DOWN	2.42	4.13E-02	DOWN
NM_001024964	Exoc3	1.34	8.76E-03	DOWN	1.54	1.98E-02	DOWN
NM_001106432	Exosc8	1.29	4.13E-02	DOWN	1.30	3.01E-02	DOWN
NM_001025406	Exosc9	1.29	4.70E-02	DOWN	1.66	7.84E-03	DOWN
NM_001107751	Ext2	1.33	3.08E-02	DOWN	1.47	4.70E-02	DOWN
NM_001100704	Extl2	1.50	4.35E-04	DOWN	1.44	2.05E-02	DOWN
NM_024132	Faah	1.42	6.08E-03	DOWN	2.87	2.97E-02	DOWN
NM_013068	Fabp2	1.32	2.38E-02	DOWN	1.83	4.38E-03	DOWN
NM_017181	Fah	1.39	1.29E-02	DOWN	2.66	3.45E-02	DOWN
NM_001134834	Fahd2a	1.52	2.62E-03	DOWN	1.73	1.46E-02	DOWN
NM_001127451	Fam103a1	1.27	2.78E-02	DOWN	1.80	2.95E-03	DOWN
NM_001191816	Fam120a	1.20	4.70E-02	DOWN	1.52	1.97E-02	DOWN
NM_001107466	Fam120b	1.25	3.01E-02	DOWN	1.69	6.76E-03	DOWN
NM_001166586	Fam122b	1.24	3.79E-02	DOWN	1.34	6.85E-03	DOWN
NM_001013878	Fam149b1	1.32	1.25E-02	DOWN	1.55	2.52E-02	DOWN
NM_001029903	Fam162a	1.48	7.83E-03	DOWN	1.68	4.54E-03	DOWN
NM_001108494	Fam168a	1.43	1.29E-02	DOWN	1.40	3.78E-02	DOWN
NM_001106401	Fam172a	1.26	4.67E-03	DOWN	1.49	1.21E-02	DOWN
NM_001106122	Fam188a	1.35	2.27E-02	DOWN	2.35	1.67E-02	DOWN
NM_001170408	Fam193b	1.34	4.74E-02	DOWN	1.75	1.98E-02	DOWN
NM_001012238	Fam20c	1.62	3.92E-02	DOWN	2.11	6.09E-03	DOWN
NM_001007688	Fam210a	1.27	5.47E-03	DOWN	1.62	7.91E-03	DOWN

NM_001106547	Fam210b	1.80	5.40E-03	DOWN	2.20	2.17E-02	DOWN
NM_001106838	Fam214a	1.20	4.74E-02	DOWN	1.53	4.88E-03	DOWN
NM_001013920	Fam227b	1.58	2.65E-03	DOWN	1.37	1.21E-02	DOWN
NM_001127681	Fam45a	1.38	1.94E-02	DOWN	1.52	1.42E-02	DOWN
NM_001170573	Fam50a	1.39	3.98E-02	DOWN	1.73	1.80E-03	DOWN
NM_001025118	Fam63a	1.49	1.33E-02	DOWN	1.92	2.53E-03	DOWN
NM_001008327	Fam96a	1.31	2.81E-03	DOWN	1.75	2.70E-03	DOWN
NM_001191718	Fance	1.58	1.28E-03	DOWN	1.65	3.08E-02	DOWN
NM_001013139	Fars2	1.38	1.13E-02	DOWN	1.65	1.11E-02	DOWN
NM_012558	Fbp1	1.45	3.04E-02	DOWN	2.88	2.66E-02	DOWN
NM_001037770	Fbxo22	1.52	2.05E-02	DOWN	1.55	3.02E-02	DOWN
NM_001014239	Fbxo25	1.29	4.68E-02	DOWN	1.53	2.24E-02	DOWN
NM_001109606	Fbxo3	1.37	5.06E-03	DOWN	1.45	3.37E-02	DOWN
NM_001107672	Fbxo4	1.27	4.30E-02	DOWN	1.85	8.32E-03	DOWN
NM_001012050	Fbxo8	1.29	2.02E-02	DOWN	2.14	1.84E-03	DOWN
NM_001025730	Fbxw5	1.56	1.69E-03	DOWN	1.56	2.73E-02	DOWN
NM_001191931	Fcrlb	1.07	3.71E-02	UP	1.08	2.90E-02	UP
NM_024153	Fdxr	1.49	2.09E-03	DOWN	1.94	1.95E-02	DOWN
NM_001108434	Fech	1.36	2.67E-02	DOWN	1.83	4.43E-03	DOWN
NM_001106928	Fer	1.37	1.00E-02	DOWN	1.39	1.90E-02	DOWN
NM_001013932	Fggy	1.49	3.84E-04	DOWN	1.48	3.39E-02	DOWN
NM_172334	Fibp	1.41	4.46E-03	DOWN	1.53	8.55E-03	DOWN
NM_001047096	Fig4	1.19	3.13E-02	DOWN	1.94	2.83E-03	DOWN
NM_001106484	Fign	1.59	4.22E-02	DOWN	1.55	2.30E-02	DOWN
NM_001191863	Fkbp4	1.38	4.81E-02	DOWN	2.03	1.45E-03	DOWN
NM_001110138	Flad1	1.51	2.25E-02	DOWN	1.45	2.74E-02	DOWN
NM_053832	Foxj1	1.08	3.06E-02	DOWN	1.26	4.50E-02	UP
NM_001290133	Frg1	1.31	3.76E-02	DOWN	1.67	3.11E-03	DOWN
NM_012848	Fth1	1.49	1.38E-02	DOWN	1.88	7.37E-03	DOWN
NM_001107125	Ftsj2	1.59	1.11E-03	DOWN	1.85	1.18E-03	DOWN
NM_001004218	Fuca2	1.49	1.74E-03	DOWN	1.69	4.98E-03	DOWN
NM_001191952	Fxn	1.41	2.46E-02	DOWN	1.57	1.06E-02	DOWN
NM_001100504	Gadd45gip1	1.47	1.52E-02	DOWN	1.60	7.71E-03	DOWN
NM_001007704	Galm	1.50	1.08E-02	DOWN	2.11	4.81E-02	DOWN
NM_001013089	Galt	2.41	1.06E-04	DOWN	2.26	1.22E-02	DOWN
NM_012793	Gamt	1.40	3.24E-02	DOWN	2.03	3.24E-02	DOWN
NM_001145840	Ganc	1.19	3.38E-02	DOWN	1.56	8.01E-03	DOWN
NM_001108339	Gatc	1.34	1.60E-02	DOWN	1.58	1.94E-02	DOWN
NM_001024277	Gcat	1.63	2.36E-03	DOWN	1.98	4.55E-02	DOWN
NM_001107633	Gcc2	1.31	1.32E-02	DOWN	1.55	1.85E-02	DOWN
NM_001108896	Gcdh	1.38	3.52E-02	DOWN	2.22	3.79E-02	DOWN

NM_172091	Gcgr	1.36	3.33E-02	DOWN	2.55	3.81E-02	DOWN
NM_133595	Gchfr	1.42	2.51E-02	DOWN	2.57	2.84E-02	DOWN
NM_001168664	Gcn1l1	1.50	3.35E-03	DOWN	1.66	1.28E-02	DOWN
NM_031749	Gcs1	1.33	3.79E-02	DOWN	1.73	3.32E-02	DOWN
NM_133598	Gcsh	1.34	1.01E-02	DOWN	2.31	2.46E-02	DOWN
NM_017276	Gdi2	1.29	1.16E-02	DOWN	1.64	6.59E-03	DOWN
NM_053389	Gemin2	1.42	7.52E-03	DOWN	1.45	3.08E-02	DOWN
NM_012566	Gfi1	1.22	1.92E-02	DOWN	1.27	1.60E-03	DOWN
NM_001100665	Gfm2	1.20	4.53E-02	DOWN	1.48	4.38E-03	DOWN
NM_001100519	Gga2	1.52	3.95E-02	DOWN	1.96	1.92E-02	DOWN
NM_001191651	Ghdc	1.38	3.19E-02	DOWN	1.63	3.47E-02	DOWN
NM_001113782	Gimd1	2.29	4.84E-03	UP	3.17	2.25E-03	DOWN
NM_001106190	Gins2	1.40	4.06E-02	DOWN	1.52	1.31E-02	DOWN
NM_024381	Gk	2.50	5.67E-03	DOWN	2.80	4.25E-02	DOWN
NM_001108820	Gla	1.31	1.75E-02	DOWN	1.80	2.91E-02	DOWN
NM_207594	Glo1	1.31	4.63E-02	DOWN	1.57	2.93E-02	DOWN
NM_001108722	Glrx5	1.37	2.87E-02	DOWN	2.12	2.15E-03	DOWN
NM_172335	Gm2a	1.36	8.40E-03	DOWN	2.31	2.79E-02	DOWN
NM_001025056	Gmppa	1.48	4.05E-03	DOWN	1.61	4.61E-02	DOWN
NM_001013036	Gmpr2	1.36	1.43E-02	DOWN	1.86	1.86E-02	DOWN
NM_053660	Gng10	1.40	1.20E-02	DOWN	1.98	9.70E-03	DOWN
NM_017084	Gnmt	2.06	2.88E-02	DOWN	3.82	1.53E-02	DOWN
NM_001007731	Golga7	1.31	1.24E-02	DOWN	1.63	9.00E-03	DOWN
NM_001109070	Golt1a	1.67	3.30E-02	DOWN	2.61	1.54E-02	DOWN
NM_001107631	Gopc	1.31	2.87E-02	DOWN	1.55	3.34E-02	DOWN
NM_013177	Got2	1.45	2.57E-03	DOWN	1.63	1.07E-02	DOWN
NM_022865	Gphn	1.35	3.41E-02	DOWN	1.85	1.21E-02	DOWN
NM_001127572	Gpn1	1.38	1.92E-02	DOWN	1.65	1.12E-02	DOWN
NM_001139486	Gpr89b	1.30	2.88E-02	DOWN	1.88	3.02E-02	DOWN
NM_030846	Grb2	1.42	3.95E-03	DOWN	1.84	6.74E-03	DOWN
NM_001198725.1	Grcc10	1.82	1.08E-02	DOWN	1.53	2.38E-02	DOWN
NM_019282	Grem1	1.23	1.12E-02	UP	1.66	3.80E-02	UP
NM_030829	Grk5	1.31	4.22E-03	DOWN	1.34	3.22E-02	DOWN
NM_001106840	Gsta4	1.51	1.67E-02	DOWN	1.78	4.90E-02	DOWN
NM_177426	Gstm2	1.79	5.61E-03	DOWN	1.87	2.44E-02	DOWN
NM_031154	Gstm7	1.65	4.09E-04	DOWN	1.99	1.41E-02	DOWN
NM_001109445	Gstz1	1.41	1.11E-02	DOWN	2.80	2.92E-02	DOWN
NM_001107318	Gtf2e2	1.30	4.21E-02	DOWN	1.82	2.59E-02	DOWN
NM_001077428	Gtf2h2	1.36	1.41E-02	DOWN	1.70	2.47E-03	DOWN
NM_001024236	Gtf2h3	1.23	4.70E-02	DOWN	1.37	4.52E-02	DOWN
NM_212501	Gtf2h4	1.51	9.76E-03	DOWN	1.74	1.01E-02	DOWN

NM_001126088	Gtf2h5	1.39	2.39E-02	DOWN	1.65	9.66E-03	DOWN
NM_001001512	Gtf2i	1.40	1.71E-02	DOWN	1.78	8.58E-03	DOWN
NM_001001504	Gtf2ird1	1.53	4.73E-02	DOWN	1.50	3.71E-02	DOWN
NM_001025120	Gtf3c2	1.35	2.07E-02	DOWN	1.55	1.68E-02	DOWN
NM_001100815	Gtpbp10	1.46	2.02E-03	DOWN	1.59	2.04E-02	DOWN
NM_012770	Gucy1b2	1.81	2.68E-03	DOWN	3.01	2.58E-02	DOWN
NM_001107215	Guf1	1.36	2.40E-02	DOWN	1.58	1.80E-02	DOWN
NM_022220	Gulo	1.36	1.74E-02	DOWN	3.12	1.77E-02	DOWN
NM_017015	Gusb	1.58	3.90E-04	DOWN	1.71	4.33E-02	DOWN
NM_017182	H2afy	1.33	2.05E-02	DOWN	1.54	4.67E-02	DOWN
NM_001135807	H2afy2	1.31	2.47E-02	DOWN	1.56	7.68E-03	DOWN
NM_053985	H3f3b	1.35	2.84E-02	DOWN	1.72	1.64E-02	DOWN
NM_053493	Hacl1	1.90	6.72E-03	UP	5.22	2.25E-03	DOWN
NM_057186	Hadh	1.38	3.01E-02	DOWN	2.27	7.34E-03	DOWN
NM_017159	Hal	2.11	1.56E-03	DOWN	2.76	3.69E-02	DOWN
NM_001191732	Hccs	1.38	3.24E-02	DOWN	1.70	6.59E-03	DOWN
NM_001100492	Hcfc1r1	1.50	4.34E-03	DOWN	1.58	2.61E-02	DOWN
NM_001005900	Hcst	1.42	7.30E-03	DOWN	1.38	2.58E-02	DOWN
NM_001035000	Hdac10	1.43	9.06E-04	DOWN	1.46	3.27E-02	DOWN
NM_001126373	Hdac8	1.43	5.27E-04	DOWN	1.56	8.10E-03	DOWN
NM_133548	Hdgfrp2	1.48	9.32E-03	DOWN	1.85	6.63E-04	DOWN
NM_001109511	Hdhd3	2.29	4.41E-04	DOWN	3.29	2.13E-03	DOWN
NM_001191064	Heatr5b	1.45	1.21E-02	DOWN	1.48	1.04E-02	DOWN
NM_001106371	Hells	1.41	1.42E-02	DOWN	1.86	1.01E-02	DOWN
NM_001012074	Herc4	1.25	4.24E-02	DOWN	1.76	1.30E-02	DOWN
NM_001142562	Hexdc	1.43	1.48E-02	DOWN	1.92	4.45E-03	DOWN
NM_001025136	Hexim1	1.37	4.33E-02	DOWN	1.84	5.52E-03	DOWN
NM_001107054	Hexim2	1.34	2.43E-02	DOWN	1.57	1.48E-02	DOWN
NM_001012145	Hgd	1.94	1.99E-03	DOWN	2.80	3.65E-02	DOWN
NM_022243	Hibadh	1.27	1.36E-02	DOWN	2.01	1.55E-02	DOWN
NM_001100825	Hint3	1.30	1.73E-02	DOWN	1.51	1.59E-02	DOWN
NM_001134763	Hip1r	1.52	3.93E-02	DOWN	1.78	4.02E-02	DOWN
NM_022647	Hist1h2bl	1.15	4.99E-02	DOWN	1.15	4.07E-02	DOWN
NM_001106114	Hist1h2bo	1.23	6.48E-03	DOWN	1.21	5.01E-03	DOWN
NM_013168	Hmbs	1.34	3.95E-02	DOWN	1.70	1.42E-02	DOWN
NM_001013184	Hmgn1	1.41	1.89E-03	DOWN	1.63	2.16E-02	DOWN
NM_001025624	Hmgn2	1.38	4.71E-02	DOWN	1.70	3.44E-02	DOWN
NM_001111294	Hnrnpa3	1.44	1.85E-02	DOWN	1.51	4.04E-02	DOWN
NM_057141	Hnrnpk	1.25	4.51E-02	DOWN	1.50	2.23E-02	DOWN
NM_001134760	Hnrnpl	1.24	1.80E-02	DOWN	1.61	1.00E-02	DOWN
NM_024404	Hnrpd	1.93	2.50E-02	DOWN	1.90	3.01E-02	DOWN

NM_152849	Homez	1.76	2.66E-04	DOWN	1.64	8.15E-03	DOWN
NM_199108	Hp1bp3	1.28	3.12E-02	DOWN	1.51	1.99E-02	DOWN
NM_173119	Hsbp1	1.33	2.96E-02	DOWN	1.81	5.93E-03	DOWN
NM_212529	Hsd17b8	1.36	2.56E-02	DOWN	1.64	1.41E-02	DOWN
NM_012584	Hsd3b5	1.50	6.20E-03	DOWN	3.22	1.71E-02	DOWN
NM_001108259	Htatsf1	1.20	3.49E-02	DOWN	1.51	1.93E-02	DOWN
NM_001106599	Htra2	1.32	1.02E-02	DOWN	1.47	1.91E-02	DOWN
NM_024357	Htt	1.23	2.71E-02	DOWN	1.47	1.43E-02	DOWN
NM_001191656	Ict1	1.38	4.00E-02	DOWN	1.58	1.74E-02	DOWN
NM_175582	Id4	1.77	4.76E-03	DOWN	2.30	3.03E-02	DOWN
NM_001037362	Idnk	1.25	6.94E-03	DOWN	1.66	3.17E-02	DOWN
NM_001172084	Idua	1.42	2.54E-02	DOWN	1.54	3.87E-02	DOWN
NM_212505	Ier3	1.86	2.43E-02	DOWN	1.88	4.36E-02	DOWN
NM_001108647	Iffo1	1.34	3.48E-02	DOWN	1.84	1.08E-02	DOWN
NM_001009625	Ifi35	1.53	2.27E-02	DOWN	2.04	4.13E-02	DOWN
NM_001105893	Ifnar1	1.37	3.03E-02	DOWN	1.56	4.19E-02	DOWN
NM_001130495	Ift27	1.45	3.96E-02	DOWN	1.73	4.32E-03	DOWN
NM_001177685	Ift52	1.47	1.66E-03	DOWN	1.73	5.14E-03	DOWN
NM_001013120	Igsf11	1.47	2.95E-02	DOWN	2.09	1.20E-02	DOWN
NM_001005537	Ik	1.36	1.45E-02	DOWN	1.57	1.81E-02	DOWN
NM_139116	Il11ra1	1.85	5.92E-04	DOWN	1.94	2.17E-03	DOWN
NM_001170604	Il12rb1	1.24	2.46E-03	DOWN	1.18	2.33E-02	DOWN
NM_001107883	Il17ra	1.39	3.21E-02	DOWN	2.32	2.28E-02	DOWN
NM_133380	Il4r	1.48	1.26E-02	DOWN	2.15	8.25E-03	DOWN
NM_133409	Ilk	1.33	1.50E-02	DOWN	1.66	2.47E-02	DOWN
NM_001108738	Ilvbl	1.45	2.90E-02	DOWN	1.77	2.28E-02	DOWN
NM_001009700	Imp4	1.30	2.32E-02	DOWN	1.66	1.11E-02	DOWN
NM_001013900	Ino80e	1.40	3.85E-02	DOWN	1.60	2.46E-02	DOWN
NM_001013859	Inpp5k	1.24	1.76E-02	DOWN	1.48	4.12E-02	DOWN
NM_001134416	Ints10	1.41	3.34E-03	DOWN	1.53	7.34E-03	DOWN
NM_001191629	Ints4	1.54	6.62E-04	DOWN	1.44	4.26E-02	DOWN
NM_001107180	Ipo9	1.34	3.89E-02	DOWN	1.39	4.79E-02	DOWN
NM_001014230	Iqcg	1.41	2.24E-02	DOWN	2.35	8.06E-03	DOWN
NM_001025422	Irak2	1.45	2.35E-02	DOWN	1.69	3.02E-02	DOWN
NM_001006969	Irf3	1.38	2.52E-02	DOWN	1.95	1.03E-02	DOWN
NM_001105936	Iscu	1.41	1.42E-02	DOWN	1.91	3.33E-03	DOWN
NM_001014242	Isoc1	1.36	2.33E-03	DOWN	2.00	2.40E-02	DOWN
NM_001008367	Isoc2b	1.50	3.03E-03	DOWN	2.18	3.56E-03	DOWN
NM_031046	Itpr2	1.31	2.73E-02	DOWN	2.03	1.56E-02	DOWN
NM_012592	Ivd	1.40	1.26E-03	DOWN	1.78	1.46E-02	DOWN
NM_001033894	Jakmip1	1.16	3.38E-02	DOWN	1.58	2.49E-02	UP

NM_001114656	Jmjd7	1.49	1.71E-02	DOWN	1.46	2.28E-02	DOWN
NM_001014116	Jmjd8	1.32	1.50E-02	DOWN	1.55	2.74E-02	DOWN
NM_001037197	Kank1	1.40	4.73E-02	DOWN	1.80	3.46E-03	DOWN
NM_001270413	Kank2	1.88	1.37E-02	DOWN	1.83	1.32E-02	DOWN
NM_001015037	Kat3	1.26	4.18E-02	DOWN	2.99	1.50E-02	DOWN
NM_001024746	Katnb1	1.60	5.88E-05	DOWN	1.34	2.34E-02	DOWN
NM_001107199	Kctd3	1.23	2.72E-02	DOWN	1.57	5.96E-03	DOWN
NM_001128194	Kctd7	1.43	1.07E-02	DOWN	1.50	1.27E-03	DOWN
NM_001108871	Kctd9	1.50	1.82E-02	DOWN	1.52	1.35E-02	DOWN
NM_001017385	Kdelr1	1.26	3.15E-02	DOWN	1.61	1.96E-02	DOWN
NM_001013122	Kdelr2	1.29	1.80E-02	DOWN	1.65	3.29E-02	DOWN
NM_001004268	Kdf1	1.43	6.37E-04	DOWN	1.51	4.85E-02	DOWN
NM_001107177	Kdm5b	1.37	8.29E-03	DOWN	1.59	9.89E-03	DOWN
NM_001108829	Kdm6b	1.49	2.08E-02	DOWN	1.57	3.93E-02	DOWN
NM_198752	Kifc2	1.80	1.75E-02	DOWN	2.64	4.46E-03	DOWN
NM_001009601	Klc4	1.38	2.90E-02	DOWN	1.79	1.78E-02	DOWN
NM_001017511	Klhl36	1.40	3.47E-03	DOWN	1.50	3.02E-02	DOWN
NM_001047093	Klhl5	1.45	2.13E-03	DOWN	1.68	1.33E-02	DOWN
NM_001107944	Klhl9	1.28	1.07E-02	DOWN	1.45	3.84E-02	DOWN
NM_001100851	Kmt2e	1.21	2.07E-02	DOWN	1.45	1.37E-02	DOWN
NM_053483	Kpna2	1.63	3.00E-02	DOWN	2.79	9.80E-05	DOWN
NM_001106444	Krtcap2	1.42	5.15E-03	DOWN	1.75	2.14E-02	DOWN
NM_001025143	Kxd1	1.39	1.05E-02	DOWN	1.60	2.67E-02	DOWN
NM_053902	Kynu	2.72	3.39E-03	DOWN	4.00	1.73E-02	DOWN
NM_001108028	L2hgdh	1.34	1.45E-02	DOWN	1.84	2.51E-03	DOWN
NM_212513	Lag3	1.56	3.72E-03	DOWN	2.21	2.87E-04	DOWN
NM_199102	Lamtor1	1.24	4.63E-02	DOWN	1.42	4.95E-02	DOWN
NM_001106462	Lamtor5	1.32	1.95E-02	DOWN	1.67	8.55E-03	DOWN
NM_001014187	Lancl2	1.20	3.12E-02	DOWN	1.28	2.79E-02	DOWN
NM_001044290	Larp7	1.33	1.53E-02	DOWN	1.49	2.10E-02	DOWN
NM_001008893	Ldhd	1.35	2.41E-02	DOWN	1.55	3.78E-02	DOWN
NM_001109271	Ldlrap1	1.59	4.58E-02	DOWN	1.99	2.71E-02	DOWN
NM_001106218	Leng1	1.58	3.19E-03	DOWN	1.64	1.02E-02	DOWN
NM_001037790	Leng8	1.43	6.95E-03	DOWN	1.43	4.70E-02	DOWN
NM_001005548	Leo1	1.44	1.21E-02	DOWN	1.75	1.00E-03	DOWN
NM_001013188	Leprotl1	1.37	1.64E-02	DOWN	1.67	1.57E-02	DOWN
NM_053862	Lgals8	1.49	3.11E-02	DOWN	2.20	1.08E-02	DOWN
NM_001012037	Lias	1.49	7.70E-04	DOWN	1.72	1.16E-02	DOWN
NM_001012011	Lig3	1.58	1.87E-02	DOWN	1.66	2.86E-02	DOWN
NM_001025715	Limd2	1.42	5.54E-03	DOWN	1.53	4.59E-02	DOWN
NM_001012163	Lims2	1.87	6.01E-04	DOWN	1.79	6.87E-03	DOWN

NM_001106245	Lin37	1.33	2.65E-02	DOWN	1.58	7.46E-03	DOWN
NM_001105735	Litaf	1.71	7.67E-03	DOWN	1.73	3.52E-02	DOWN
NM_001115024	Lman2	1.40	4.79E-03	DOWN	1.80	8.98E-03	DOWN
NM_001079939	Lmf2	1.26	3.08E-02	DOWN	1.60	2.20E-02	DOWN
NM_001103356	LOC100125364	1.29	1.77E-02	DOWN	1.83	1.58E-02	DOWN
NM_001139487	LOC100125368	1.64	3.93E-02	DOWN	1.77	4.58E-02	DOWN
NM_001127606	LOC100158225	1.47	2.18E-02	DOWN	2.06	1.42E-02	DOWN
NM_001131003	LOC100174910	1.31	1.14E-02	DOWN	1.69	8.15E-03	DOWN
NM_001142941	LOC100233176	1.42	2.01E-02	DOWN	1.62	1.93E-02	DOWN
NM_001270388	LOC100360619	2.05	1.67E-02	DOWN	2.12	2.88E-02	DOWN
NM_001177816	LOC100361645	1.56	2.78E-02	DOWN	3.38	4.11E-03	DOWN
NM_001177819	LOC100362110	1.44	2.28E-02	DOWN	2.75	4.63E-03	DOWN
NM_001271975	LOC100365921	1.50	1.00E-02	DOWN	2.05	9.17E-04	DOWN
NM_001277222	LOC100911483	1.34	3.69E-02	DOWN	1.54	3.85E-02	DOWN
NR_110377	LOC102723236	10.91	2.82E-02	DOWN	10.87	2.72E-02	DOWN
NR_110639	LOC287004	1.23	1.15E-02	DOWN	1.26	1.31E-02	DOWN
NM_001134533	LOC301124	1.31	3.84E-02	DOWN	1.72	1.81E-02	DOWN
NM_001037190	LOC303448	1.50	3.12E-02	DOWN	2.26	8.97E-04	DOWN
NM_001013981	LOC304396	1.37	2.88E-02	DOWN	1.48	1.51E-02	DOWN
NM_001134574	LOC361646	1.31	1.76E-02	DOWN	1.79	1.11E-02	DOWN
NM_001109058	LOC498122	1.34	2.49E-02	DOWN	1.54	4.96E-02	DOWN
NM_001166307	LOC498592	1.61	1.70E-02	DOWN	2.17	8.62E-03	DOWN
NM_001024308	LOC499742	1.61	2.82E-02	DOWN	1.88	2.75E-02	DOWN
NM_001100869	LOC499781	1.28	2.79E-02	DOWN	1.29	3.81E-02	DOWN
NM_001047958	LOC500594	1.12	1.50E-02	DOWN	1.14	1.35E-03	DOWN
NM_022271	LOC64038	1.50	2.37E-02	DOWN	1.91	1.09E-02	DOWN
NM_001037658	LOC652955	1.31	2.87E-02	DOWN	2.04	3.88E-03	DOWN
NM_001101006	LOC683077	1.32	1.95E-02	DOWN	1.58	1.33E-02	DOWN
NM_001145273	LOC688869	1.38	3.98E-02	DOWN	1.56	2.84E-02	DOWN
NM_001127573	LOC689226	1.28	1.67E-02	DOWN	1.74	3.18E-03	DOWN
NM_001109581	LOC690349	1.46	9.87E-03	DOWN	1.95	2.05E-03	DOWN
NM_001195482	LOC690871	1.39	4.57E-03	DOWN	1.64	3.55E-02	DOWN
NM_001198796	LOC691849	1.39	2.12E-02	DOWN	1.97	1.77E-03	DOWN
NM_001135566	LOC691921	1.43	4.85E-02	DOWN	1.50	4.37E-02	DOWN
NM_133404	Lonp1	1.25	1.74E-02	DOWN	1.64	8.66E-03	DOWN
NM_001191585	Lonrf3	1.93	3.08E-02	UP	2.05	1.50E-02	DOWN
NM_001109376	Lpgat1	1.32	4.00E-02	DOWN	2.13	4.08E-02	DOWN
NM_001108555	Lrba	1.26	8.46E-03	DOWN	1.70	1.08E-02	DOWN
NM_001127551	Lrch4	1.60	2.90E-03	DOWN	1.49	4.25E-02	DOWN
NM_001106321	Lrp5	1.90	5.40E-03	DOWN	1.81	5.24E-03	DOWN
NM_001108486	Lrrc28	1.26	4.33E-02	DOWN	1.84	5.80E-03	DOWN

NM_001009710	Lrrc41	1.32	3.31E-02	DOWN	1.52	3.12E-02	DOWN
NM_001025653	Lrrc42	1.45	1.33E-02	DOWN	1.50	2.07E-02	DOWN
NM_001008280	Lrrc59	1.54	1.20E-02	DOWN	1.76	3.60E-02	DOWN
NM_001109231	Lrrc61	1.72	4.08E-03	DOWN	1.94	1.32E-02	DOWN
NM_001107701	Lrrc71	1.34	4.99E-02	DOWN	1.77	7.62E-03	DOWN
NM_001107204	Lrrc8b	1.39	1.83E-02	DOWN	1.86	1.49E-02	DOWN
NM_001008338	Lrrc8d	1.61	1.31E-02	DOWN	2.15	1.25E-02	DOWN
NM_001108976	Lsm10	1.42	2.74E-02	DOWN	1.88	3.58E-03	DOWN
NM_001107289	Lsm5	1.29	3.40E-02	DOWN	1.75	2.12E-03	DOWN
NM_001108732	Lsm7	1.24	4.60E-02	DOWN	1.56	1.91E-02	DOWN
NM_001008315	Ltbr	1.51	3.41E-02	DOWN	1.99	3.17E-02	DOWN
NM_001107853	Luc7l2	1.40	3.65E-02	DOWN	1.76	4.23E-03	DOWN
NM_013006	Lypla1	1.26	4.60E-02	DOWN	2.14	7.08E-04	DOWN
NM_031342	Lypla2	1.38	1.07E-02	DOWN	1.72	1.70E-02	DOWN
NM_001105986	Lyplal1	1.43	2.61E-03	DOWN	1.87	1.90E-02	DOWN
NM_001126096	Lyrm2	1.26	3.20E-02	DOWN	1.71	2.55E-03	DOWN
NM_001134729	Lyrm7	1.35	1.50E-02	DOWN	1.94	2.53E-02	DOWN
NM_001024302	Lysmd1	1.29	8.36E-04	DOWN	1.58	9.37E-03	DOWN
NM_153470	Lzts1	1.72	3.80E-02	DOWN	1.75	3.22E-02	DOWN
NM_001106594	Mad2l1	1.40	2.55E-02	DOWN	1.41	1.45E-02	DOWN
NM_001012106	Mad2l2	1.42	4.06E-02	DOWN	1.42	2.98E-02	DOWN
NM_001008319	Maea	1.29	1.92E-02	DOWN	1.40	3.30E-02	DOWN
NM_001013250	Mageh1	1.53	6.22E-04	DOWN	1.43	7.80E-03	DOWN
NM_139256	Man2c1	1.34	8.16E-03	DOWN	1.37	3.99E-02	DOWN
NM_001173380	Manbal	1.33	2.73E-02	DOWN	1.75	1.60E-02	DOWN
NM_133283	Map2k2	1.34	4.39E-02	DOWN	1.90	3.07E-03	DOWN
NM_133407	Map4k3	1.29	1.12E-02	DOWN	1.45	3.03E-02	DOWN
NM_001135716	Map9	1.49	2.46E-02	DOWN	1.44	4.32E-02	DOWN
NM_001109532	Mapk11	1.23	4.41E-02	DOWN	1.33	5.18E-03	DOWN
NM_017322	Mapk9	1.48	1.82E-02	DOWN	2.24	1.37E-02	DOWN
NM_133421.1	Marf1	1.30	2.20E-02	DOWN	1.98	6.37E-03	DOWN
NM_001134796	Mast3	1.77	1.30E-03	DOWN	1.64	4.10E-02	DOWN
NM_001109056	Mb21d2	1.37	3.13E-02	DOWN	1.60	3.02E-02	DOWN
NM_001170566	Mbd6	1.41	3.02E-03	DOWN	1.53	1.61E-02	DOWN
NM_012599	Mbl1	1.41	4.16E-03	DOWN	2.73	4.91E-02	DOWN
NM_001109120	Mboat1	1.53	3.47E-02	DOWN	1.95	7.12E-03	DOWN
NM_001012177	Mccc2	1.32	8.75E-03	DOWN	1.69	1.90E-02	DOWN
NM_001106341	Mcee	1.33	2.26E-02	DOWN	2.02	1.57E-03	DOWN
NM_139253	Mcfd2	1.37	6.04E-03	DOWN	2.25	3.94E-02	DOWN
NM_019323	Mcpt9	1.39	4.35E-02	DOWN	1.69	1.86E-03	DOWN
NM_001017459	Mdm1	1.54	1.36E-05	DOWN	1.46	5.72E-03	DOWN

NM_001106039	Mdp1	1.69	3.00E-03	DOWN	1.54	2.06E-02	DOWN
NM_001044286	Mea1	1.39	2.11E-02	DOWN	1.58	2.02E-02	DOWN
NM_022673	Mecp2	1.34	6.21E-04	DOWN	1.43	3.54E-02	DOWN
NM_001106097	Med10	1.34	1.96E-03	DOWN	1.41	2.48E-02	DOWN
NM_001105799	Med11	1.39	2.28E-03	DOWN	1.40	4.78E-02	DOWN
NM_001107217	Med28	1.32	5.26E-03	DOWN	1.65	1.18E-02	DOWN
NM_001106237	Med29	1.52	1.66E-02	DOWN	1.67	4.82E-03	DOWN
NM_001130539	Med30	1.34	7.67E-03	DOWN	1.45	1.08E-02	DOWN
NM_001024256	Med4	1.67	3.77E-03	DOWN	1.84	1.30E-02	DOWN
NM_001108673	Med8	1.29	4.20E-02	DOWN	1.80	1.92E-03	DOWN
NM_001017507	Mef2b	1.15	1.65E-02	DOWN	1.15	2.50E-02	DOWN
NM_001107940	Megf9	1.63	2.17E-02	DOWN	1.61	2.79E-02	DOWN
NM_001115033	Meiob	1.43	4.96E-02	DOWN	2.87	1.26E-04	DOWN
NM_001134702	Meis1	1.39	1.81E-03	DOWN	1.66	9.91E-03	DOWN
NM_017149	Meox2	1.57	3.64E-02	DOWN	2.01	1.81E-02	DOWN
NM_001025668	Mettl18	1.23	2.13E-02	DOWN	1.89	1.78E-02	DOWN
NM_001271284	Mff	1.26	3.81E-02	DOWN	2.08	2.31E-02	DOWN
NM_001106911	Mfsd6	1.32	3.66E-02	DOWN	2.29	1.07E-02	DOWN
NM_001108215	Mfsd9	1.50	6.15E-03	DOWN	2.18	2.49E-03	DOWN
NM_030861	Mgat1	1.36	1.29E-02	DOWN	1.76	4.75E-02	DOWN
NM_001024895	MGC114492	1.15	3.74E-02	DOWN	1.73	2.37E-02	UP
NM_001024905	MGC116121	1.17	1.33E-02	DOWN	1.47	2.89E-02	DOWN
NM_001077231	MGC125239	1.24	1.72E-02	DOWN	2.09	1.37E-03	DOWN
NR_027366	MGC94282	1.66	1.59E-02	DOWN	1.58	3.00E-02	DOWN
NM_001004251	MGC94335	1.29	1.96E-02	DOWN	1.68	8.50E-03	DOWN
NM_001005532	MGC95210	1.21	4.84E-02	DOWN	1.60	1.62E-02	DOWN
NM_001009655	Mgme1	1.42	8.81E-03	DOWN	1.38	2.14E-02	DOWN
NM_001106430	Mgst2	1.26	2.17E-02	DOWN	1.85	4.84E-02	DOWN
NM_134399	Mk1	1.61	8.79E-03	DOWN	1.93	3.60E-02	DOWN
NM_001008353	Mkks	1.37	4.87E-03	DOWN	1.72	1.89E-03	DOWN
NM_001034917	Mks1	1.60	1.25E-02	DOWN	1.63	1.50E-02	DOWN
NM_001108105	Mlc1	1.52	4.53E-02	DOWN	2.65	2.48E-02	DOWN
NM_001107889	Mlf2	1.29	2.64E-02	DOWN	1.61	1.05E-02	DOWN
NM_133552	Mlxipl	1.23	4.94E-02	DOWN	2.42	3.42E-02	DOWN
NM_001106174	Mmaa	1.44	8.63E-03	DOWN	1.87	1.50E-03	DOWN
NM_080776	Mmp16	1.23	2.72E-02	DOWN	1.30	1.29E-02	DOWN
NM_001107159	Mmp19	1.31	3.66E-02	DOWN	2.36	1.77E-02	DOWN
NM_001271596	Mms19l	1.48	4.68E-03	DOWN	1.36	3.70E-02	DOWN
NM_001106881	Mocs1	1.70	2.09E-03	DOWN	1.83	1.06E-02	DOWN
NM_001011999	Morf4l1	1.28	3.06E-02	DOWN	1.66	2.06E-02	DOWN
NM_001025629	Mospd3	1.53	3.54E-03	DOWN	2.02	2.34E-02	DOWN

NM_133561	Mpc1	1.37	4.10E-02	DOWN	2.17	1.95E-03	DOWN
NM_001108435	Mppe1	1.40	7.17E-03	DOWN	1.89	1.11E-02	DOWN
NM_053814	Mprip	1.38	9.87E-03	DOWN	1.49	3.11E-02	DOWN
NM_138843	Mpst	1.69	1.32E-03	DOWN	1.90	7.43E-03	DOWN
NM_001098240	Mpv17l	1.35	4.51E-02	DOWN	1.91	6.56E-03	DOWN
NM_001192002	Mreg	1.71	3.33E-02	DOWN	2.07	8.74E-03	DOWN
NM_001010947	Mri1	1.33	4.91E-02	DOWN	1.54	2.52E-02	DOWN
NM_001105997.1	Mrpl1	1.43	8.58E-03	DOWN	1.66	5.30E-04	DOWN
NM_001109620	Mrpl10	1.34	1.60E-02	DOWN	1.78	3.97E-03	DOWN
NM_001006973	mrpl11	1.29	3.76E-02	DOWN	1.79	5.07E-03	DOWN
NM_001271131	Mrpl14	1.56	3.70E-03	DOWN	1.61	1.78E-02	DOWN
NM_001106633	Mrpl15	1.32	8.78E-03	DOWN	1.71	4.41E-03	DOWN
NM_001009647	Mrpl16	1.30	2.40E-02	DOWN	1.65	7.25E-03	DOWN
NM_001029898	Mrpl19	1.49	3.64E-03	DOWN	1.67	1.02E-02	DOWN
NM_001034136	Mrpl2	1.41	4.79E-03	DOWN	1.59	1.06E-02	DOWN
NM_001109428	Mrpl20	1.43	2.89E-02	DOWN	2.12	1.91E-03	DOWN
NM_022529	Mrpl23	1.41	1.54E-02	DOWN	1.48	2.93E-02	DOWN
NM_001106596	Mrpl35	1.30	4.46E-02	DOWN	1.62	1.07E-02	DOWN
NM_001108879	Mrpl36	1.31	4.35E-02	DOWN	1.73	7.63E-03	DOWN
NM_001004235	Mrpl37	1.29	1.76E-02	DOWN	1.61	1.04E-02	DOWN
NM_001108754	Mrpl4	1.30	3.07E-02	DOWN	1.69	2.17E-02	DOWN
NM_001013426	Mrpl41	1.36	2.11E-02	DOWN	1.54	4.20E-02	DOWN
NM_001013068	Mrpl46	1.31	4.00E-02	DOWN	1.66	9.46E-03	DOWN
NM_001047883	Mrpl49	1.49	2.34E-02	DOWN	1.42	4.31E-02	DOWN
NM_001108665	Mrpl50	1.36	2.90E-02	DOWN	1.77	2.73E-03	DOWN
NM_001106621	Mrpl51	1.32	1.51E-02	DOWN	1.43	2.95E-02	DOWN
NM_001108635	Mrpl53	1.38	1.87E-02	DOWN	1.60	9.17E-03	DOWN
NM_001109148	Mrps11	1.33	3.91E-02	DOWN	1.76	4.41E-03	DOWN
NM_001106239	Mrps12	1.38	1.69E-02	DOWN	1.86	5.10E-03	DOWN
NM_001109518	Mrps16	1.52	8.44E-03	DOWN	1.71	5.77E-03	DOWN
NM_001105923	Mrps17	1.28	2.29E-02	DOWN	1.78	4.78E-03	DOWN
NM_198756	Mrps18a	1.24	4.53E-02	DOWN	1.55	1.42E-02	DOWN
NM_212534	Mrps18b	1.42	2.83E-02	DOWN	1.52	3.76E-02	DOWN
NM_001077530	Mrps22	1.43	6.80E-03	DOWN	1.55	2.42E-02	DOWN
NM_001108289	Mrps23	1.32	3.41E-02	DOWN	1.87	3.44E-03	DOWN
NM_001077659	Mrps24	1.32	1.90E-02	DOWN	1.54	2.33E-02	DOWN
NM_001013206	Mrps26	1.36	3.22E-02	DOWN	1.78	1.14E-02	DOWN
NM_001047909	Mrps28	1.27	3.57E-02	DOWN	1.44	9.21E-03	DOWN
NM_001106412	Mrps30	1.45	4.29E-04	DOWN	1.50	8.24E-03	DOWN
NM_001106091	Mrps31	1.40	2.41E-02	DOWN	1.61	1.13E-02	DOWN
NM_001008354	Mrrf	1.33	1.24E-02	DOWN	1.45	3.51E-02	DOWN

NM_031058	Msh2	1.45	2.55E-02	DOWN	1.49	3.84E-02	DOWN
NM_001031660	Msrb2	1.59	1.53E-04	DOWN	2.02	2.51E-02	DOWN
NM_001100740	Mta2	1.28	5.00E-02	DOWN	1.60	2.36E-02	DOWN
NM_001009697	Mtfmt	1.43	3.80E-03	DOWN	1.72	2.97E-03	DOWN
NM_001009349	Mthfs	1.42	5.33E-03	DOWN	2.14	2.23E-02	DOWN
NM_001004254	Mtif2	1.37	2.65E-02	DOWN	1.63	1.79E-02	DOWN
NM_001115041	Mtif3	1.32	1.75E-02	DOWN	1.59	2.11E-02	DOWN
NM_001013047	Mtm1	1.42	4.43E-03	DOWN	1.40	3.84E-02	DOWN
NM_001105827	Mtmr4	1.38	1.91E-02	DOWN	1.98	9.14E-03	DOWN
NM_001100667	Mtx1	1.36	9.72E-03	DOWN	1.68	4.22E-03	DOWN
NM_199375	Mvb12a	1.40	9.43E-03	DOWN	1.75	1.32E-02	DOWN
NM_183332	Myadm	1.42	2.90E-02	DOWN	1.43	3.96E-02	UP
NM_017343	Myl12b	1.34	2.49E-02	DOWN	1.73	1.30E-02	DOWN
NM_001191592	N6amt1	1.35	1.23E-02	DOWN	1.69	5.85E-04	DOWN
NM_001105794	Naa38	1.30	4.84E-02	DOWN	1.79	7.54E-03	DOWN
NM_001014216	Nabp1	1.56	1.05E-02	DOWN	1.84	6.45E-03	DOWN
NM_001244819	Nabp2	1.45	2.94E-02	DOWN	1.52	3.64E-02	DOWN
NM_001109678	Nadk	1.22	4.70E-02	DOWN	1.85	3.57E-02	DOWN
NM_001044252	Nadk2	1.22	4.43E-02	DOWN	2.38	1.65E-02	DOWN
NM_181480	Nadsyn1	1.45	1.72E-02	DOWN	1.82	8.85E-03	DOWN
NM_001012120	Naga	1.40	3.45E-03	DOWN	1.80	2.81E-02	DOWN
NM_001107053	Nags	1.56	1.36E-02	DOWN	2.49	4.70E-02	DOWN
NM_207609	Naprt1	1.45	8.00E-03	DOWN	1.81	4.36E-02	DOWN
NM_001039207	Narf	1.38	3.63E-02	DOWN	1.73	7.73E-03	DOWN
NM_001037316	Nat1	1.27	2.25E-02	DOWN	2.79	2.64E-03	DOWN
NM_022635	Nat8	3.46	1.12E-02	UP	4.45	2.33E-03	DOWN
NM_001014082	Ncln	1.32	4.41E-02	DOWN	1.69	3.42E-02	DOWN
NM_001107818	Ndor1	1.53	1.99E-02	DOWN	1.77	1.33E-02	DOWN
NM_001270863	Ndrg2	1.42	4.53E-03	DOWN	1.80	1.93E-02	DOWN
NM_182671	Ndufa10l1	1.37	6.90E-03	DOWN	1.59	4.11E-03	DOWN
NM_212517	Ndufa11	1.30	4.70E-02	DOWN	1.62	1.82E-02	DOWN
NM_001106781	Ndufa12	1.31	3.13E-02	DOWN	1.51	2.20E-02	DOWN
NM_001100752	Ndufa9	1.36	5.32E-03	DOWN	1.56	3.89E-02	DOWN
NM_001106500	Ndufaf1	1.32	3.18E-02	DOWN	1.53	1.22E-02	DOWN
NM_001033971	Ndufaf3	1.89	2.29E-02	DOWN	1.86	8.04E-03	DOWN
NM_001008318	Ndufaf7	1.39	1.68E-02	DOWN	1.45	1.65E-02	DOWN
NM_001109443	Ndufb10	1.48	6.92E-03	DOWN	1.72	3.42E-02	DOWN
NM_001106756	Ndufb11	1.34	2.99E-02	DOWN	1.65	1.85E-02	DOWN
NM_001108624	Ndufb2	1.40	3.73E-02	DOWN	1.55	3.53E-02	DOWN
NM_001106912	Ndufb3	1.40	3.13E-02	DOWN	1.83	1.01E-02	DOWN
NM_001106646	Ndufb6	1.33	1.66E-02	DOWN	1.78	8.23E-03	DOWN

NM_001106322	Ndufs8	1.46	1.15E-02	DOWN	1.52	4.20E-02	DOWN
NM_001006972	Ndufv1	1.32	2.43E-02	DOWN	1.55	3.89E-02	DOWN
NM_031064	Ndufv2	1.28	3.07E-02	DOWN	1.53	3.17E-02	DOWN
NM_022607	Ndufv3	1.50	3.47E-02	DOWN	1.66	2.83E-02	DOWN
NM_199096	Necap2	1.27	4.56E-02	DOWN	1.49	3.43E-02	DOWN
NM_138878	Nedd8	1.30	3.59E-02	DOWN	1.67	1.43E-02	DOWN
NM_001025754	Neil1	1.34	3.17E-02	DOWN	1.68	2.32E-03	DOWN
NM_053462	Nfs1	1.20	2.27E-02	DOWN	1.72	3.56E-02	DOWN
NM_012866	Nfyc	1.32	7.62E-03	DOWN	1.43	2.81E-02	DOWN
NM_001100730	Nipsnap1	1.43	3.87E-03	DOWN	2.66	3.47E-02	DOWN
NM_182668	Nit1	1.53	6.61E-03	DOWN	2.55	4.79E-03	DOWN
NM_001034126	Nit2	1.48	6.59E-03	DOWN	2.48	1.08E-02	DOWN
NM_001034148	Nmi	1.57	4.19E-02	DOWN	1.98	1.58E-02	DOWN
NM_001037556	Nmnat1	1.51	1.24E-02	DOWN	1.86	1.27E-03	DOWN
NM_001126100	Nop10	1.34	4.11E-02	DOWN	1.99	4.42E-03	DOWN
NM_001106260	Nosip	1.42	1.70E-02	DOWN	1.67	1.78E-02	DOWN
NM_001013975	Notum	2.01	2.91E-02	DOWN	3.30	2.60E-02	DOWN
NM_153624	Npc1	1.68	1.90E-02	DOWN	1.92	4.32E-02	DOWN
NM_001191882	Nphp3	1.44	2.02E-02	DOWN	1.45	1.40E-02	DOWN
NM_001004214	Nqo2	1.61	3.10E-03	DOWN	1.98	3.77E-02	DOWN
NM_031626	Nr1h2	1.34	1.70E-02	DOWN	1.50	4.52E-02	DOWN
NM_031627	Nr1h3	1.46	2.39E-02	DOWN	2.44	2.47E-02	DOWN
NM_080778	Nr2f2	1.61	1.07E-02	DOWN	2.03	3.43E-03	DOWN
NM_139113	Nr2f6	1.49	6.94E-03	DOWN	1.97	1.32E-02	DOWN
NM_022186	Nrbf2	1.41	1.13E-02	UP	1.80	1.71E-02	DOWN
NM_001034997	Nrbp1	1.32	4.51E-02	DOWN	1.50	2.77E-02	DOWN
NM_001191109	Nrg4	2.39	1.40E-02	UP	1.89	1.95E-02	DOWN
NM_001135006	Nrn1l	1.13	3.52E-03	DOWN	1.16	6.55E-04	DOWN
NM_207607	Ns5atp4	1.49	4.10E-02	DOWN	1.54	4.33E-02	DOWN
NM_031981	Nsfl1c	1.32	4.37E-02	DOWN	1.51	4.04E-02	DOWN
NM_181389	Nsmaf	1.19	3.78E-02	DOWN	1.60	4.44E-03	DOWN
NM_001039611	Nsmce1	1.34	3.70E-02	DOWN	1.56	2.08E-02	DOWN
NM_001024876	Nsmce2	1.34	2.65E-02	DOWN	1.67	1.11E-02	DOWN
NR_073057	Nsmf	1.46	1.78E-02	DOWN	1.41	1.79E-02	DOWN
NM_001107862	Nt5c3a	1.39	2.16E-02	DOWN	1.57	1.08E-02	DOWN
NM_001106393	Nt5dc1	1.40	4.03E-02	DOWN	2.34	4.49E-03	DOWN
NM_001025124	Ntan1	1.33	1.41E-02	DOWN	1.93	1.14E-03	DOWN
NM_001105728	Nthl1	1.40	1.67E-02	DOWN	1.61	2.21E-02	DOWN
NM_001011891	Nubp2	1.48	2.90E-02	DOWN	1.67	1.29E-02	DOWN
NM_001185025	Nubpl	1.34	2.24E-03	DOWN	1.55	1.38E-02	DOWN
NM_057120	Nudt1	1.21	4.77E-02	DOWN	1.57	7.04E-03	DOWN

NM_001106760	Nudt14	1.45	1.30E-02	DOWN	1.48	4.23E-02	DOWN
NM_001100732	Nudt18	1.59	2.68E-03	DOWN	1.52	2.48E-02	DOWN
NM_199090	Nudt22	1.76	5.01E-03	DOWN	1.53	1.56E-02	DOWN
NM_001007733	Nudt5	1.31	4.99E-02	DOWN	1.58	3.19E-02	DOWN
NM_001168559	Nup214	1.38	2.30E-02	DOWN	1.48	3.87E-02	DOWN
NM_001134570	Nupr1l	1.48	1.47E-02	DOWN	1.61	6.03E-03	DOWN
NM_022521	Oat	2.09	4.95E-03	DOWN	2.17	1.91E-02	DOWN
NM_001013874	Ociad1	1.25	4.96E-02	DOWN	1.64	1.21E-02	DOWN
NM_001271181	Ociad2	1.52	3.39E-02	DOWN	1.87	2.36E-02	DOWN
NM_001108256	Ocrl	1.47	1.09E-03	DOWN	1.60	7.81E-03	DOWN
NM_001106961	Ofd1	1.35	1.97E-02	DOWN	1.60	1.18E-02	DOWN
NM_030870	Ogg1	1.29	3.75E-02	DOWN	1.49	2.58E-02	DOWN
NM_001000081	Olr1584	1.15	2.29E-02	DOWN	1.17	6.92E-03	DOWN
NM_001106669	Oma1	1.42	5.76E-03	DOWN	1.56	3.56E-03	DOWN
NM_199092	Orc4	1.32	3.35E-02	DOWN	1.69	1.89E-03	DOWN
NM_001014186	Orc5	1.28	2.41E-02	DOWN	1.63	7.97E-03	DOWN
NM_001105940	Ormdl2	1.29	1.37E-02	DOWN	2.11	2.20E-02	DOWN
NM_172023	Osbpl1a	1.32	5.93E-03	DOWN	1.49	1.30E-02	DOWN
NM_001107044	Osbpl7	1.67	1.33E-04	DOWN	1.37	4.73E-02	DOWN
NM_001005384	Osmr	1.43	3.82E-02	UP	1.40	2.61E-02	UP
NM_001106332	Otub1	1.33	4.18E-02	DOWN	1.61	1.23E-02	DOWN
NM_001197332	Oxr1	1.26	3.21E-02	DOWN	1.56	1.67E-02	DOWN
NM_001100508	Oxsm	1.35	6.28E-03	DOWN	1.73	7.34E-04	DOWN
NM_001135008	Pabpn1	1.37	2.68E-02	DOWN	1.73	9.32E-03	DOWN
NM_001024898	Paf1	1.34	3.55E-02	DOWN	1.61	1.05E-02	DOWN
NM_001008862	Pan2	1.20	3.21E-02	DOWN	1.80	8.18E-03	DOWN
NM_001106375	Papss2	1.61	4.81E-04	DOWN	2.90	8.01E-03	DOWN
NM_001277250	Park7	1.36	2.33E-02	DOWN	1.70	1.21E-02	DOWN
NM_013063	Parp1	1.28	1.85E-02	DOWN	1.43	4.47E-02	DOWN
NM_001106030	Parp2	1.30	9.39E-03	DOWN	1.58	8.44E-03	DOWN
NM_001014064	Pars2	1.34	2.12E-02	DOWN	1.73	8.81E-03	DOWN
NM_001277215	Patz1	1.64	3.52E-02	DOWN	1.63	1.12E-02	DOWN
NM_019125	Pbsn	1.27	1.52E-02	DOWN	1.26	9.47E-03	DOWN
NM_001100681	Pbx1	1.50	3.67E-03	DOWN	1.80	3.80E-03	DOWN
NM_019330	Pcca	1.35	1.86E-02	DOWN	1.71	2.50E-02	DOWN
NM_017030	Pccb	1.34	5.71E-03	DOWN	1.67	1.43E-02	DOWN
NM_001037153	Pcdhga11	1.09	4.73E-02	DOWN	1.10	1.68E-02	DOWN
NM_001039454	Pced1b	1.34	2.77E-02	DOWN	1.83	1.18E-02	DOWN
NM_001108377	Pck2	1.44	3.26E-02	DOWN	1.97	1.89E-03	DOWN
NM_053823	Pcsk5	1.29	8.98E-03	DOWN	1.84	2.02E-04	DOWN
NM_001009542	Pdcd10	1.25	3.81E-02	DOWN	1.68	3.42E-03	DOWN

NM_031525	Pdgfrb	1.62	2.19E-05	DOWN	1.52	3.32E-02	DOWN
NM_017319	Pdia3	1.32	2.81E-02	DOWN	2.06	3.33E-02	DOWN
NM_017062	Pdlim4	1.43	3.81E-03	DOWN	2.28	6.35E-03	UP
NM_001107430	Pdpr	1.75	5.10E-03	DOWN	1.63	8.47E-03	DOWN
NM_001014249	Pdss2	1.31	3.26E-02	DOWN	1.56	3.68E-02	DOWN
NM_001106945	Pdzd11	1.29	4.55E-02	DOWN	1.74	8.59E-03	DOWN
NM_031712	Pdzk1	1.74	9.02E-04	DOWN	2.95	1.07E-02	DOWN
NM_001109405	Pex10	1.36	2.94E-02	DOWN	1.54	2.28E-02	DOWN
NM_001105902	Pex11g	1.71	1.50E-03	DOWN	1.99	1.13E-03	DOWN
NM_001012088	Pex16	1.35	2.75E-02	DOWN	2.23	1.07E-02	DOWN
NM_017234	Pex2	1.44	4.40E-03	DOWN	1.75	7.04E-03	DOWN
NM_001170584	Pex5	1.39	4.58E-03	DOWN	2.18	5.63E-03	DOWN
NM_212506	Pfdn6	1.40	4.49E-02	DOWN	2.00	2.47E-03	DOWN
NM_022511	Pfn1	1.36	3.37E-02	DOWN	1.61	2.42E-02	DOWN
NM_053895	Pgap2	1.40	3.55E-02	DOWN	1.51	4.66E-02	DOWN
NM_001143895	Pgap3	1.43	9.26E-03	DOWN	1.53	3.69E-02	DOWN
NM_001106066	Pgls	1.36	3.31E-02	DOWN	1.67	3.51E-02	DOWN
NM_001013035	Phb2	1.37	9.64E-03	DOWN	1.68	1.42E-02	DOWN
NM_001110492	Phf14	1.22	4.11E-02	DOWN	1.53	2.30E-02	DOWN
NM_001128196	Phykpl	1.35	2.01E-02	DOWN	1.91	3.77E-02	DOWN
NM_053337	Pias2	1.22	7.75E-03	DOWN	1.73	5.38E-04	DOWN
NM_001108714	Pigh	1.29	3.74E-02	DOWN	1.71	1.33E-02	DOWN
NM_001011953	Pigk	1.34	3.70E-03	DOWN	1.79	6.16E-03	DOWN
NM_001099758	Pigp	1.38	1.37E-02	DOWN	1.44	2.24E-02	DOWN
NM_001010966	Pigv	1.36	2.24E-02	DOWN	1.44	2.92E-03	DOWN
NM_001024370	Pigy	1.31	3.29E-02	DOWN	1.63	6.76E-03	DOWN
NM_001109525	Pigz	1.32	2.99E-02	DOWN	1.61	4.95E-03	DOWN
NM_022185	Pik3r2	1.34	6.78E-03	DOWN	1.47	3.39E-02	DOWN
NM_001033970	Pip5k1c	1.29	2.16E-02	DOWN	1.62	2.51E-02	DOWN
NM_001012009	Pipox	1.36	1.62E-02	DOWN	2.99	4.40E-02	DOWN
NM_001108007	Pkdcc	1.61	1.63E-02	DOWN	1.94	3.07E-02	DOWN
NM_001107764	Pla2g4b	1.59	1.37E-03	DOWN	1.53	1.88E-02	DOWN
NM_001013927	Plbd1	1.36	3.38E-02	DOWN	1.68	3.16E-02	DOWN
NM_030992	Pld1	1.53	8.38E-03	DOWN	1.76	2.77E-02	DOWN
NM_001108072	Plekhj1	1.34	2.62E-02	DOWN	1.62	1.63E-02	DOWN
NM_001134613	Plekhs1	1.53	2.54E-03	DOWN	1.71	1.86E-02	DOWN
NM_001106347	Plgrkt	1.63	1.55E-04	DOWN	1.74	1.09E-04	DOWN
NM_001007144	Plin2	1.74	1.04E-02	UP	2.21	1.81E-02	DOWN
NM_001109068	Pm20d1	1.81	1.18E-04	DOWN	2.20	4.49E-02	DOWN
NM_001106973	Pmm2	1.51	6.09E-03	DOWN	1.57	1.32E-02	DOWN
NM_022395	Pmpcb	1.37	6.00E-03	DOWN	1.53	1.61E-02	DOWN

NM_001025274	Pnisr	1.27	4.38E-02	DOWN	1.63	1.09E-02	DOWN
NM_001134751	Pnkd	2.24	1.96E-04	DOWN	1.45	4.71E-02	DOWN
NM_001103360	Pnrc2	1.28	1.66E-02	DOWN	1.41	3.45E-02	DOWN
NM_001105816	Poldip2	1.31	3.29E-02	DOWN	1.57	2.45E-02	DOWN
NM_001106002	Polr2b	1.32	2.90E-03	DOWN	1.45	1.09E-02	DOWN
NM_031335	Polr2f	1.38	1.21E-02	DOWN	1.42	4.68E-02	DOWN
NM_001105921	Polr2j	1.43	1.82E-02	DOWN	1.66	1.32E-02	DOWN
NM_001109571	Polr3gl	1.26	1.24E-03	DOWN	1.67	4.73E-03	DOWN
NM_001106766	Polrmt	1.33	3.86E-02	DOWN	1.70	1.12E-02	DOWN
NM_001024883	Pomk	1.35	9.97E-03	DOWN	1.51	7.92E-03	DOWN
NM_053406	Pomt1	1.47	3.93E-03	DOWN	1.66	6.94E-03	DOWN
NM_032077	Pon1	1.29	1.38E-02	DOWN	3.08	2.58E-02	DOWN
NM_001130550	Pop1	1.24	3.43E-02	DOWN	1.49	2.41E-02	DOWN
NM_001009642	Pop4	1.57	1.02E-02	DOWN	1.60	1.11E-02	DOWN
NM_001024322	Pot1	1.29	1.12E-02	DOWN	1.46	2.42E-02	DOWN
NM_001105746	Pou6f1	1.44	2.95E-02	DOWN	1.35	4.39E-02	DOWN
NM_001134567	Pp2d1	1.41	4.51E-02	DOWN	1.63	3.31E-03	DOWN
NM_001135871	Ppa2	1.36	2.28E-02	DOWN	2.14	4.36E-03	DOWN
NM_001034854	Ppapdc2	1.40	4.75E-03	DOWN	1.67	4.67E-02	DOWN
NM_001009316	Ppdpf	1.61	1.39E-03	DOWN	2.19	8.13E-03	DOWN
NM_001108745	Ppfia2	1.20	2.85E-02	DOWN	1.44	1.53E-02	DOWN
NM_001100582	Ppfibp2	1.32	3.54E-02	DOWN	1.62	2.37E-02	DOWN
NM_001108992	Pphln1	1.22	4.45E-02	DOWN	1.73	1.63E-03	DOWN
NM_017101	Ppia	1.26	4.96E-02	DOWN	1.74	2.28E-02	DOWN
NM_022536	Ppib	1.42	1.02E-02	DOWN	1.83	4.75E-02	DOWN
NM_001017383	Ppil2	1.38	1.60E-02	DOWN	1.71	5.76E-03	DOWN
NM_175707	Ppil3	1.54	5.79E-03	DOWN	1.73	8.51E-03	DOWN
NM_001270620	Ppm1b	1.59	3.04E-02	DOWN	1.51	8.00E-03	DOWN
NM_001105968	Ppox	1.64	3.22E-04	DOWN	1.73	1.24E-02	DOWN
NM_212542	Ppp1r11	1.54	3.43E-03	DOWN	1.57	1.64E-02	DOWN
NM_001130566	Ppp1r16a	1.58	1.72E-02	DOWN	1.98	4.04E-03	DOWN
NM_001271305	Ppp1r21	1.46	1.25E-03	DOWN	1.47	1.39E-02	DOWN
NM_001012072	Ppp1r3c	2.88	1.59E-03	DOWN	1.82	4.51E-02	DOWN
NM_001009825	Ppp1r7	1.31	6.69E-03	DOWN	1.53	5.71E-03	DOWN
NM_001014196	Ppp2r3c	1.25	2.12E-02	DOWN	1.81	1.68E-03	DOWN
NM_001108577	Ppp2r4	1.30	4.41E-02	DOWN	1.50	3.12E-02	DOWN
NM_001107891	Ppp2r5a	1.38	1.28E-02	DOWN	1.60	3.02E-02	DOWN
NM_001135849	Ppp6r1	1.29	4.31E-02	DOWN	1.44	4.62E-02	DOWN
NM_001106281	Prcp	1.70	1.60E-02	DOWN	2.45	6.04E-03	DOWN
NM_022540	Prdx3	1.38	3.24E-03	DOWN	1.52	4.11E-02	DOWN
NM_053512	Prdx4	1.29	4.11E-02	DOWN	2.13	4.01E-02	DOWN

NM_001009636	Prelid1	1.40	2.01E-02	DOWN	2.13	2.57E-02	DOWN
NM_001271333	Prelid2	1.73	1.19E-03	UP	2.08	2.50E-02	DOWN
NM_013010	Prkag1	1.33	5.67E-03	DOWN	1.48	1.81E-02	DOWN
NM_184051	Prkag2	1.50	8.33E-03	DOWN	1.65	2.41E-02	DOWN
NM_001024263	Prkd3	1.47	1.70E-02	DOWN	2.04	4.36E-02	DOWN
NM_001024780	Prkra	1.40	2.59E-02	DOWN	1.64	3.09E-02	DOWN
NM_057131	Prpsap2	1.39	1.42E-02	DOWN	1.36	4.88E-02	DOWN
NM_001108432	Prr16	1.52	4.49E-02	DOWN	2.70	2.97E-02	DOWN
NM_181477	Prss21	1.28	3.19E-03	DOWN	1.19	2.61E-02	DOWN
NM_175765	Psip1	1.36	1.96E-02	DOWN	1.66	6.25E-03	DOWN
NM_017278	Psma1	1.38	3.81E-02	DOWN	1.72	1.25E-02	DOWN
NM_017279	Psma2	1.38	4.29E-02	DOWN	1.84	3.46E-03	DOWN
NM_017285	Psmb3	1.35	3.23E-02	DOWN	1.54	1.32E-02	DOWN
NM_057123	Psmc1	1.32	3.08E-02	DOWN	1.67	1.66E-02	DOWN
NM_001025689	Psmd14	1.42	3.41E-02	DOWN	1.64	1.84E-02	DOWN
NM_130430	Psmd9	1.79	2.10E-03	DOWN	2.03	3.20E-03	DOWN
NM_017264	Psme1	1.30	3.08E-02	DOWN	1.60	4.74E-02	DOWN
NM_001101005	Psmf1	1.31	4.22E-02	DOWN	2.02	3.93E-03	DOWN
NM_001105891	Psmg1	1.37	1.91E-02	DOWN	2.27	5.46E-04	DOWN
NM_001106138	Psmg2	1.28	2.97E-02	DOWN	1.58	7.78E-03	DOWN
NM_001009679	Psph	1.41	4.20E-02	DOWN	1.63	1.67E-02	DOWN
NM_001134718	Ptcd3	1.24	3.64E-02	DOWN	1.55	2.08E-02	DOWN
NM_013081	Ptk2	1.35	1.02E-02	DOWN	1.48	2.09E-02	DOWN
NM_031975	Ptms	1.47	8.40E-03	DOWN	2.35	9.21E-03	DOWN
NM_001106831	Ptplad1	1.50	1.50E-02	DOWN	2.37	6.98E-03	DOWN
NM_001105726	Ptpmt1	1.29	4.43E-02	DOWN	1.56	1.18E-02	DOWN
NM_019249	Ptprf	1.55	4.22E-03	DOWN	2.64	5.87E-03	DOWN
NM_019140	Ptprs	1.29	3.58E-02	DOWN	1.41	3.31E-02	DOWN
NM_001191880	Puf60	1.38	2.79E-02	DOWN	1.52	3.54E-02	DOWN
NM_001011993	Pycrl	1.56	3.12E-03	DOWN	2.11	1.61E-03	DOWN
NM_022390	Qdpr	1.37	3.78E-02	DOWN	2.14	4.75E-02	DOWN
NM_001009646	Qprt	1.40	1.47E-02	DOWN	2.43	4.00E-02	DOWN
NM_001130557	R3hdm2	1.30	2.16E-02	DOWN	1.57	4.31E-02	DOWN
NM_031152	Rab11a	1.28	3.72E-02	DOWN	1.54	1.31E-02	DOWN
NM_001109979	Rab1b	1.36	2.03E-02	DOWN	1.50	4.48E-02	DOWN
NM_001015012	Rab30	1.60	2.70E-02	UP	2.85	1.13E-02	DOWN
NM_001108944	Rab33b	1.32	2.32E-02	DOWN	1.81	8.48E-03	DOWN
NM_001012140	Rab34	1.45	2.28E-02	DOWN	1.51	2.28E-02	DOWN
NM_145774	Rab38	1.82	1.42E-03	DOWN	1.88	9.27E-03	DOWN
NM_017313	Rab3ip	1.29	2.05E-02	DOWN	1.99	3.51E-02	DOWN
NM_013019	Rab4a	1.47	9.30E-03	DOWN	1.70	5.60E-03	DOWN

NM_023950	Rab7a	1.17	3.70E-02	DOWN	1.64	7.49E-03	DOWN
NM_053998	Rab8a	1.35	2.67E-02	DOWN	1.93	1.71E-02	DOWN
NM_019124	Rabep1	1.44	4.62E-03	DOWN	1.57	6.53E-03	DOWN
NM_001024871	Rabepk	1.41	6.16E-04	DOWN	1.60	2.08E-03	DOWN
NM_031654	Rabggta	1.30	1.09E-02	DOWN	1.70	8.26E-03	DOWN
NM_001007678	Rabif	1.27	4.03E-03	DOWN	1.51	3.50E-02	DOWN
NM_001013221	Rabl2a	1.25	3.88E-02	DOWN	1.75	1.18E-02	DOWN
NM_001191755	Rad54b	1.20	3.53E-02	UP	1.09	4.55E-02	DOWN
NM_053439	Ran	1.36	1.53E-02	DOWN	1.38	3.18E-02	DOWN
NM_001106950	Rap2c	1.39	8.08E-03	DOWN	1.59	5.12E-03	DOWN
NM_031528	Rara	1.40	8.77E-04	DOWN	1.70	4.17E-02	DOWN
NM_021764	Rbck1	1.44	8.13E-03	DOWN	1.46	4.81E-02	DOWN
NM_001107373	Rbfa	1.49	9.23E-04	DOWN	2.00	1.63E-04	DOWN
NM_001277160	Rbm26	1.28	2.61E-02	DOWN	1.43	1.62E-02	DOWN
NM_153306	Rbm45	1.27	3.48E-02	DOWN	1.55	1.25E-02	DOWN
NM_001025663	Rbmx	1.37	4.65E-03	DOWN	1.46	2.10E-02	DOWN
NM_001034135	Rbx1	1.30	2.05E-02	DOWN	1.74	1.14E-02	DOWN
NM_001100585	Rce1	1.21	3.63E-02	DOWN	1.72	1.02E-02	DOWN
NM_001105842	Rdm1	1.47	4.86E-03	DOWN	1.61	4.64E-02	DOWN
NM_001005889	Rdx	1.33	3.19E-02	DOWN	1.86	1.39E-03	DOWN
NM_001105853	Recql5	1.44	2.70E-02	DOWN	1.70	8.90E-03	DOWN
NM_001025279	Reep4	1.37	4.02E-02	DOWN	1.53	4.78E-02	DOWN
NM_001008326	Rexo2	1.30	3.29E-02	DOWN	1.85	1.13E-03	DOWN
NM_001105869	Rfc4	1.22	2.38E-02	DOWN	1.55	7.67E-03	DOWN
NM_001135866	Rft1	1.41	3.22E-04	DOWN	1.40	3.21E-02	DOWN
NM_001127490	Rfx7	1.33	1.99E-02	DOWN	1.48	4.17E-02	DOWN
NM_001013136	Rfxank	1.42	3.85E-02	DOWN	1.62	2.85E-02	DOWN
NM_001004225	RGD1303003	1.33	2.23E-02	DOWN	1.60	4.35E-02	DOWN
NM_001134547	RGD1304770	1.58	7.14E-03	DOWN	1.86	3.51E-02	DOWN
NM_001106730	RGD1305089	1.27	3.44E-02	DOWN	1.66	1.27E-02	DOWN
NM_001127452	RGD1305587	1.48	3.64E-03	DOWN	2.05	3.56E-02	DOWN
NM_001106026	RGD1306063	1.60	1.14E-03	DOWN	1.70	1.64E-02	DOWN
NM_001108746	RGD1306474	1.53	1.69E-02	DOWN	1.57	7.18E-03	DOWN
NM_001037188	RGD1307155	1.44	1.37E-02	DOWN	1.79	1.01E-02	DOWN
NM_001134508	RGD1307603	2.00	1.84E-02	DOWN	4.00	1.60E-02	DOWN
NM_001134575	RGD1308106	1.33	4.43E-02	DOWN	1.33	2.78E-02	DOWN
NM_001127521	RGD1308134	1.34	6.33E-03	DOWN	1.57	4.42E-03	DOWN
NM_001106491	RGD1309730	1.63	1.60E-03	DOWN	1.60	1.21E-02	DOWN
NM_001035517	RGD1310127	1.34	2.02E-03	DOWN	1.59	3.14E-02	DOWN
NM_001106765	RGD1310212	1.33	8.44E-03	DOWN	1.63	1.20E-02	DOWN
NM_001106999	RGD1310352	1.22	2.61E-02	DOWN	1.90	5.24E-03	DOWN

NM_001173436	RGD1311345	1.31	3.97E-02	DOWN	1.79	2.78E-02	DOWN
NM_001014075	RGD1311648	1.29	4.66E-02	DOWN	1.69	2.01E-02	DOWN
NM_001013898	RGD1311703	1.27	2.84E-02	DOWN	1.44	4.39E-02	DOWN
NM_001047898	RGD1359290	1.36	1.86E-02	DOWN	1.89	1.43E-03	DOWN
NM_001107915	RGD1559904	1.30	1.23E-02	DOWN	1.52	2.63E-02	DOWN
NM_001107685	RGD1560010	1.62	1.12E-03	UP	1.65	1.51E-03	DOWN
NM_001134616	RGD1560065	1.30	3.60E-02	DOWN	1.55	1.61E-02	DOWN
NM_001025122	RGD1561328	1.45	3.11E-02	DOWN	1.72	3.39E-03	DOWN
NM_001170475	RGD1562018	1.23	1.11E-02	DOWN	1.88	1.84E-02	DOWN
NM_001106212	RGD1562747	1.36	4.67E-02	DOWN	1.56	4.60E-02	DOWN
NM_001173472	RGD1562987	1.30	3.74E-02	DOWN	1.84	7.41E-03	DOWN
NM_001105946	RGD1564093	1.25	4.85E-02	DOWN	1.80	6.19E-03	DOWN
NM_001126295	RGD1564379	1.43	9.44E-03	DOWN	1.61	1.56E-02	DOWN
NM_001164396	RGD1564865	1.80	5.89E-03	DOWN	3.00	1.05E-02	DOWN
NM_001271394	RGD1565002	1.53	2.76E-02	DOWN	1.73	2.35E-02	DOWN
NM_001109050	RGD1565033	1.45	8.80E-04	DOWN	1.55	2.12E-02	DOWN
NM_001109101	RGD1565222	1.15	4.16E-02	DOWN	1.42	7.87E-03	DOWN
NM_001109179	RGD1565641	1.32	4.55E-02	DOWN	1.85	5.13E-04	DOWN
NM_001126286	RGD1565685	1.26	2.33E-02	DOWN	1.65	5.52E-03	DOWN
NM_001115047	RGD1566052	1.34	2.47E-02	DOWN	1.33	3.67E-02	DOWN
NM_199493	RGD735029	1.45	3.08E-03	DOWN	1.52	1.53E-02	DOWN
NM_199379	RGD735065	1.46	2.75E-02	DOWN	1.95	2.88E-02	DOWN
NM_001106805	Rgl3	1.71	2.11E-03	DOWN	1.81	4.12E-02	DOWN
NM_001077589	Rgs16	9.87	2.42E-04	UP	5.32	6.03E-03	UP
NM_001105819	Rhbdl3	1.24	2.70E-02	DOWN	3.02	1.28E-04	UP
NM_001007715	Ribc1	1.52	7.13E-03	DOWN	1.51	3.41E-02	DOWN
NM_001105811	Rilp	1.32	2.18E-02	DOWN	1.61	3.37E-02	DOWN
NM_001107786	Rin2	1.73	1.49E-02	DOWN	2.11	2.19E-02	DOWN
NM_212549	Ring1	1.43	1.70E-02	DOWN	1.43	4.55E-02	DOWN
NM_001106241	Rinl	1.47	1.09E-02	DOWN	1.66	1.14E-02	DOWN
NM_001031663	Rmdn1	1.29	3.92E-02	DOWN	2.17	7.55E-04	DOWN
NM_001037200	Rmdn2	2.41	1.54E-03	DOWN	2.32	3.58E-02	DOWN
NM_001106210	Rnaset2	1.23	4.84E-02	DOWN	1.52	4.54E-02	DOWN
NM_001107540	Rnf121	1.34	2.73E-02	DOWN	1.44	1.71E-02	DOWN
NM_001127545	Rnf139	1.28	4.64E-02	DOWN	1.77	4.00E-02	DOWN
NM_001008361	Rnf167	1.38	4.52E-03	DOWN	1.49	2.77E-02	DOWN
NM_001107234	Rnf215	1.28	1.73E-02	DOWN	1.76	3.55E-03	DOWN
NM_001113748	Rnf26	1.44	1.71E-02	DOWN	1.60	4.70E-02	DOWN
NM_001135921	Rnf43	1.93	1.18E-02	DOWN	2.06	1.04E-02	DOWN
NM_001107923	Rngtt	1.19	4.24E-02	DOWN	1.72	8.08E-03	DOWN
NM_139105	Rnh1	1.44	3.83E-02	DOWN	1.97	1.61E-02	DOWN

NM_001014167	Rnls	1.31	3.18E-02	DOWN	2.07	3.17E-02	DOWN
NM_001195490	Romo1	1.59	3.88E-02	DOWN	1.66	3.11E-02	DOWN
NM_001109012	Rp2	1.41	1.10E-02	DOWN	1.51	9.15E-03	DOWN
NM_001004243	Rpap3	1.22	1.49E-02	DOWN	1.47	1.36E-02	DOWN
NM_001130578	Rps19bp1	1.39	4.69E-03	DOWN	1.65	4.66E-03	DOWN
NM_001010962	Rps6kb2	1.34	3.56E-02	DOWN	1.64	1.98E-02	DOWN
NM_001134499	Rptor	1.25	4.55E-02	DOWN	1.47	2.17E-02	DOWN
NM_001108641	Rpusd3	1.61	6.78E-03	DOWN	1.88	4.10E-03	DOWN
NM_001109404	Rsu1	1.54	1.90E-04	DOWN	1.72	1.06E-03	DOWN
NM_001008831	RT1-Ba	1.36	3.29E-02	DOWN	2.13	3.02E-02	DOWN
NM_001008847	RT1-Da	1.44	7.48E-03	DOWN	2.21	3.06E-02	DOWN
NM_001008884	RT1-Db1	1.35	4.39E-02	DOWN	2.03	4.25E-02	DOWN
NM_012646	RT1-N1	3.03	4.18E-03	DOWN	2.78	5.41E-03	DOWN
NM_001004227	Rtcd1	1.39	3.03E-02	DOWN	1.57	2.31E-02	DOWN
NM_001033890	Rtfdc1	1.31	2.92E-02	DOWN	1.48	3.44E-02	DOWN
NM_001047116	Rundc3b	1.37	2.64E-02	DOWN	1.53	4.11E-03	DOWN
NM_147177	Ruvbl1	1.29	1.07E-02	DOWN	1.39	2.90E-02	DOWN
NM_001271173	Sbf1	1.32	2.81E-02	DOWN	1.47	3.14E-02	DOWN
NM_019384	Scaf1	1.36	1.08E-02	DOWN	1.45	4.90E-02	DOWN
NM_001013985	Sccpdh	1.27	3.01E-02	DOWN	1.72	1.52E-02	DOWN
NM_019364	Scfd1	1.51	3.32E-03	DOWN	1.51	2.76E-02	DOWN
NM_001107638	Scml4	1.61	9.92E-03	DOWN	1.89	3.38E-02	DOWN
NM_031548	Scnn1a	1.97	2.38E-02	DOWN	2.51	1.41E-02	DOWN
NM_138508	Scp2	1.59	2.39E-03	DOWN	2.68	1.51E-02	DOWN
NM_001012142	Scrn2	1.68	4.69E-04	DOWN	2.17	5.90E-03	DOWN
NM_001011938	Scyl1	1.26	4.13E-02	DOWN	1.54	1.48E-02	DOWN
NM_013082	Sdc2	1.22	2.45E-02	DOWN	2.62	3.75E-02	DOWN
NM_130412	Sdf4	1.26	1.80E-02	DOWN	1.94	1.36E-02	DOWN
NM_001008371	Sdhaf2	1.38	1.29E-02	DOWN	1.61	1.32E-02	DOWN
NM_001100539	Sdhb	1.38	1.17E-02	DOWN	1.64	1.51E-02	DOWN
NM_031723	Sec11a	1.32	1.95E-02	DOWN	1.60	3.12E-02	DOWN
NM_153628	Sec11c	1.34	3.09E-02	DOWN	1.77	2.57E-02	DOWN
NM_001006978	Sec13	1.51	6.49E-03	DOWN	1.67	5.85E-03	DOWN
NM_057147	Sec22a	1.49	3.36E-03	DOWN	1.72	4.88E-02	DOWN
NM_001106474	Sec24b	1.25	1.10E-02	DOWN	1.32	3.97E-02	DOWN
NM_001109456	Sec24c	1.35	2.64E-02	DOWN	1.67	2.12E-02	DOWN
NM_001107637	Sec63	1.28	3.05E-02	DOWN	1.91	3.19E-02	DOWN
NM_080892	Selenbp1	1.36	2.78E-02	DOWN	1.83	1.66E-02	DOWN
NM_207589	Selk	1.33	3.25E-02	DOWN	1.69	1.53E-02	DOWN
NM_001105888	Senp7	1.24	1.19E-02	DOWN	1.40	2.53E-02	DOWN
NM_001128287	Sepsecs	1.31	2.81E-02	DOWN	1.84	3.98E-02	DOWN

NM_022616	Septin 7	1.31	4.80E-02	DOWN	2.04	1.36E-03	DOWN
NM_001113497	Septin 9	1.57	5.64E-03	DOWN	1.96	8.11E-03	DOWN
NM_001130579	Serhl2	1.80	1.49E-02	DOWN	2.18	2.25E-02	DOWN
NM_177927	Serpinf1	1.36	2.09E-02	DOWN	2.53	3.96E-02	DOWN
NM_001017513	Sertad3	1.54	2.95E-02	DOWN	1.88	1.39E-02	DOWN
NM_001106167	Setd6	1.29	4.61E-02	DOWN	1.98	1.51E-03	DOWN
NM_001271175	Setdb1	1.52	1.04E-02	DOWN	1.72	1.27E-02	DOWN
NM_001105754	Sez6	1.25	4.48E-02	DOWN	6.76	1.11E-03	DOWN
NM_001025698	Sf3a3	1.40	1.10E-02	DOWN	1.46	3.79E-02	DOWN
NM_031647	Sfmbt1	1.52	9.18E-03	DOWN	1.71	1.30E-02	DOWN
NM_001034924	Sfswap	1.34	4.19E-02	DOWN	1.53	2.87E-02	DOWN
NM_001008302	Sft2d1	1.25	2.40E-02	DOWN	1.59	4.72E-02	DOWN
NM_001034011	Sft2d2	1.35	2.03E-02	DOWN	1.69	4.19E-02	DOWN
NM_134463	Sgk2	2.46	1.71E-03	DOWN	3.12	7.55E-03	DOWN
NM_001271080	Shkbp1	1.50	7.87E-03	DOWN	1.70	9.94E-03	DOWN
NM_001008322	Shmt2	1.38	2.74E-02	DOWN	2.18	1.14E-02	DOWN
NM_001108759	Siae	1.40	2.02E-03	DOWN	2.33	5.67E-03	DOWN
NM_001024887	Sigirr	1.48	6.52E-03	DOWN	2.30	3.55E-02	DOWN
NM_030996	Sigmar1	2.00	5.62E-05	DOWN	2.54	3.22E-02	DOWN
NM_199376	Sil1	1.28	3.93E-02	DOWN	1.84	4.23E-02	DOWN
NM_001115038	Sin3b	1.43	3.37E-02	DOWN	1.59	2.83E-02	DOWN
NM_001008368	Sirt2	1.31	2.74E-02	DOWN	1.60	2.17E-02	DOWN
NM_213559	Skiv2l	1.32	2.37E-02	DOWN	1.56	2.54E-02	DOWN
NM_001106416	Skp2	1.26	3.40E-02	DOWN	1.38	1.56E-02	DOWN
NM_017047	Slc10a1	1.52	3.04E-02	DOWN	3.40	2.26E-02	DOWN
NM_001025280	Slc10a5	1.37	9.81E-03	DOWN	1.48	2.50E-02	DOWN
NM_001012621	Slc13a4	2.67	3.88E-04	DOWN	5.81	1.14E-03	DOWN
NM_001013913	Slc16a4	1.54	4.71E-03	DOWN	3.86	5.99E-03	DOWN
NM_001271214	Slc17a4	1.55	1.77E-03	DOWN	2.48	4.80E-02	DOWN
NM_001030024	Slc19a2	1.52	1.08E-02	DOWN	2.63	4.86E-02	DOWN
NM_017315	Slc23a1	1.68	1.18E-03	DOWN	2.35	4.00E-02	DOWN
NM_001017488	Slc24a6	1.45	2.78E-02	DOWN	1.87	2.93E-02	DOWN
NM_139100	Slc25a3	1.33	1.87E-02	DOWN	2.09	1.74E-02	DOWN
NM_001030032	Slc25a38	1.41	4.38E-02	DOWN	2.10	8.34E-03	DOWN
NM_001024792	Slc25a39	1.35	8.67E-03	DOWN	2.12	5.90E-03	DOWN
NM_001127590	Slc25a42	1.52	3.28E-02	UP	2.01	6.40E-03	DOWN
NM_057102	Slc25a5	1.33	3.68E-02	DOWN	2.33	1.27E-02	DOWN
NM_001143817	Slc26a6	1.52	6.04E-03	DOWN	1.45	4.51E-02	DOWN
NM_031684	Slc29a1	1.81	2.60E-03	DOWN	1.78	3.82E-02	DOWN
NM_181639	Slc29a3	1.79	8.74E-04	DOWN	1.61	1.45E-02	DOWN
NM_053494	Slc2a8	1.49	7.08E-03	DOWN	1.61	1.62E-02	DOWN

NM_001105985	Slc30a10	1.35	4.56E-02	DOWN	2.01	4.78E-02	DOWN
NM_001107924	Slc35a1	1.24	1.68E-02	DOWN	1.81	5.98E-03	DOWN
NM_001106590	Slc35b4	1.32	2.23E-02	DOWN	1.47	1.54E-02	DOWN
NM_001105950	Slc35f5	1.35	1.87E-02	DOWN	1.45	1.25E-02	DOWN
NM_001008356	Slc39a3	1.44	1.55E-02	DOWN	1.60	4.45E-02	DOWN
NM_001008885	Slc39a7	1.50	4.11E-02	DOWN	1.80	4.67E-02	DOWN
NM_001011952	Slc39a8	1.73	3.81E-03	DOWN	2.68	1.65E-02	DOWN
NM_001108742	Slc41a2	1.38	1.70E-02	DOWN	2.71	3.25E-02	DOWN
NM_130746	Slc5a6	1.46	3.76E-02	DOWN	1.65	4.01E-02	DOWN
NM_017335	Slc6a12	1.37	2.80E-02	DOWN	2.43	2.56E-02	DOWN
NM_053811	Slc9a3r2	1.28	4.08E-02	DOWN	1.40	4.78E-02	DOWN
NM_031650	Slco1b2	1.99	8.03E-03	DOWN	2.80	4.79E-02	DOWN
NM_001108222	Smarcal1	1.33	1.92E-02	DOWN	1.70	2.12E-03	DOWN
NM_001024993	Smarce1	1.30	1.19E-02	DOWN	1.63	1.78E-02	DOWN
NM_031683	Smc1a	1.29	3.13E-02	DOWN	1.50	1.43E-02	DOWN
NM_001134584	Smco4	1.69	5.09E-04	DOWN	1.92	5.49E-03	DOWN
NM_001131000	Smim11	1.45	1.89E-02	DOWN	1.74	3.10E-02	DOWN
NM_001201374	Smim8	1.40	6.76E-03	DOWN	2.19	2.54E-03	DOWN
NR_051995	Smndc1	1.53	1.80E-02	DOWN	2.03	1.32E-02	DOWN
NM_001006997	Smpd1	1.41	5.29E-03	DOWN	1.90	1.05E-02	DOWN
NM_001167806	Smpd4	1.29	6.36E-03	DOWN	1.57	9.07E-03	DOWN
NM_057195	Smu1	1.32	1.23E-02	DOWN	1.44	3.10E-02	DOWN
NM_013035	Snai2	1.83	1.29E-03	DOWN	1.42	4.49E-02	DOWN
NM_001108033	Snapc1	1.41	3.45E-03	DOWN	1.63	2.85E-03	DOWN
NM_001170576	Snapin	1.37	1.32E-02	DOWN	1.50	2.26E-02	DOWN
NM_001109399	Snrpd2	1.35	3.26E-02	DOWN	1.56	2.46E-02	DOWN
NM_001004270	Snupn	1.37	2.36E-02	DOWN	1.56	1.93E-02	DOWN
NM_001109279	Snw1	1.31	4.53E-02	DOWN	1.69	7.25E-03	DOWN
NM_053411	Snx1	1.40	2.48E-03	DOWN	1.45	2.69E-02	DOWN
NM_001108131	Snx19	1.40	4.30E-02	DOWN	1.79	6.76E-03	DOWN
NM_001106518	Snx5	1.36	8.14E-03	DOWN	1.45	3.30E-02	DOWN
NM_001127637	Snx9	1.30	1.92E-02	DOWN	1.49	3.14E-02	DOWN
NM_153728	Soat2	1.53	2.20E-02	DOWN	2.57	3.07E-02	DOWN
NM_001271149	Socs6	1.23	2.09E-02	DOWN	1.54	4.26E-03	DOWN
NM_001106552	Sp5	1.33	1.00E-02	DOWN	1.44	1.32E-02	DOWN
NM_053482	Spa17	1.40	7.64E-03	DOWN	1.67	3.80E-02	DOWN
NM_001191601	Spcs2	1.40	6.51E-03	DOWN	1.59	1.98E-02	DOWN
NM_001006987	Spg21	1.44	8.45E-03	DOWN	1.90	1.74E-02	DOWN
NM_001271297	Spock1	1.34	9.31E-04	DOWN	1.56	6.15E-03	DOWN
NM_138533	Spon2	1.60	1.88E-04	DOWN	1.48	8.47E-03	DOWN
NM_001014200	Sppl2b	1.39	1.83E-02	DOWN	1.54	2.09E-02	DOWN

NM_019181	Spr	1.34	4.73E-02	DOWN	2.14	8.55E-03	DOWN
NM_001037765	Spryd4	1.56	2.11E-03	DOWN	1.84	1.51E-03	DOWN
NM_001106988	Spsb3	1.40	3.49E-02	DOWN	1.58	2.73E-02	DOWN
NM_001008373	Srek1ip1	1.30	1.67E-02	DOWN	1.55	1.99E-02	DOWN
NM_001106497	Srp14	1.25	4.95E-02	DOWN	1.75	3.13E-03	DOWN
NM_001106157	Srp19	1.37	3.74E-02	DOWN	1.59	2.30E-02	DOWN
NM_001195505	Srsf5	1.38	2.53E-02	DOWN	2.56	2.67E-03	DOWN
NM_001009255	Srsf9	1.24	8.02E-03	DOWN	1.79	8.08E-03	DOWN
NM_031119	Ssb	1.27	9.19E-03	DOWN	1.81	1.78E-03	DOWN
NM_183328	Ssbp1	1.35	2.47E-02	DOWN	2.16	3.22E-03	DOWN
NM_001107820	Ssna1	1.38	9.69E-03	DOWN	1.97	3.20E-03	DOWN
NM_031120	Ssr3	1.31	1.58E-02	DOWN	1.69	2.68E-02	DOWN
NM_017199	Ssr4	1.31	3.12E-02	DOWN	1.71	1.26E-02	DOWN
NM_207602	St3gal6	1.32	1.38E-02	DOWN	1.54	2.74E-02	DOWN
NM_001007639	St7l	1.37	3.79E-02	DOWN	1.49	3.27E-02	DOWN
NM_013029	St8sia3	1.62	3.06E-02	DOWN	2.95	7.23E-03	DOWN
NM_001108179	Stag1	1.52	3.41E-02	DOWN	1.98	7.22E-03	DOWN
NM_138531	Stambp	1.31	4.67E-02	DOWN	1.56	2.67E-02	DOWN
NM_001013069	Stard10	1.41	2.80E-02	DOWN	2.25	4.70E-02	DOWN
NM_001109060	Stard13	1.73	1.51E-02	DOWN	2.29	9.58E-03	DOWN
NM_001008298	Stard3nl	1.45	1.42E-03	DOWN	1.62	6.82E-03	DOWN
NM_017064	Stat5a	1.53	5.24E-03	DOWN	1.63	3.01E-03	DOWN
NM_001109907	Stau1	1.52	3.62E-02	DOWN	1.78	7.54E-03	DOWN
NM_173142	Stk16	1.37	1.13E-02	DOWN	2.07	2.05E-02	DOWN
NM_031735	Stk3	1.20	3.44E-02	DOWN	1.59	1.91E-03	DOWN
NM_001083336	Stk38l	1.34	5.66E-03	DOWN	1.49	1.73E-02	DOWN
NM_024346	Stmn3	1.39	5.35E-03	DOWN	1.30	2.41E-02	DOWN
NM_001031646	Stoml2	1.36	1.07E-02	DOWN	1.68	1.12E-02	DOWN
NM_012748	Stx2	1.69	8.73E-03	DOWN	2.02	7.56E-03	DOWN
NM_031656	Stx8	1.41	4.60E-03	DOWN	1.84	1.31E-03	DOWN
NM_031126	Stxbp2	1.39	1.71E-02	DOWN	1.71	4.21E-02	DOWN
NM_001100750	Suclg2	1.27	2.86E-02	DOWN	1.97	1.64E-02	DOWN
NM_133547	Sult1c2	3.34	8.51E-03	DOWN	3.45	3.89E-02	DOWN
NM_001013177	Sult1c2a	1.70	4.92E-03	DOWN	2.78	4.41E-02	DOWN
NM_001024295	Sumo3	1.38	1.37E-02	DOWN	1.55	3.98E-02	DOWN
NM_172068	Surf1	1.37	9.66E-03	DOWN	1.47	1.73E-02	DOWN
NM_001033868	Surf4	1.57	1.98E-03	DOWN	1.87	3.62E-02	DOWN
NM_001107475	Suv420h2	1.62	5.28E-04	DOWN	1.58	2.63E-02	DOWN
NM_001246661	Swi5	1.33	3.36E-02	DOWN	1.64	1.95E-02	DOWN
NM_001004253	Syap1	1.28	1.16E-02	DOWN	1.57	4.12E-03	DOWN
NM_181632	Syt15	1.26	2.34E-02	DOWN	1.40	1.09E-02	UP

NM_001108302	Taco1	1.38	3.27E-03	DOWN	1.88	4.90E-05	DOWN
NM_001025734	Tada3	1.51	2.41E-03	DOWN	1.39	4.25E-02	DOWN
NM_031811	Taldo1	1.35	6.50E-03	DOWN	1.67	1.28E-02	DOWN
NM_001106622	Tapbpl	1.67	7.88E-04	DOWN	1.70	4.39E-02	DOWN
NM_001014040	Tars2	1.48	2.08E-03	DOWN	1.42	3.87E-02	DOWN
NM_001044243	Tasp1	1.46	4.43E-02	DOWN	1.54	2.71E-02	DOWN
NM_001025748	Taz	1.36	1.30E-02	DOWN	1.55	4.15E-02	DOWN
NM_001108921	Tbc1d10b	1.37	1.33E-02	DOWN	1.46	3.77E-02	DOWN
NM_001134842	Tbc1d31	1.33	1.60E-02	DOWN	1.48	1.18E-03	DOWN
NM_001040180.1	Tbcb	1.49	3.82E-02	DOWN	2.43	2.58E-02	DOWN
NM_001012161	Tbce	1.33	4.76E-02	DOWN	1.93	8.86E-04	DOWN
NM_001025152	Tc2n	1.34	2.16E-02	DOWN	1.57	8.63E-03	DOWN
NM_001014275	Tceal8	1.37	5.04E-03	DOWN	1.45	2.08E-02	DOWN
NM_001014260	Tceanc2	1.34	3.89E-03	DOWN	1.64	1.09E-02	DOWN
NM_001270562	Tceb1	1.41	1.40E-02	DOWN	2.09	1.11E-03	DOWN
NM_001191052	Tcf7l2	1.49	2.30E-02	DOWN	1.96	5.19E-03	DOWN
NM_001191666	Tchp	1.31	4.27E-02	DOWN	1.45	3.57E-02	DOWN
NM_199089	Tcirg1	1.87	1.87E-03	DOWN	1.84	1.52E-02	DOWN
NM_001025626	Tefm	1.33	1.79E-02	DOWN	1.41	2.31E-02	DOWN
NR_001567	Terc	1.25	1.66E-03	DOWN	1.19	5.93E-03	DOWN
NM_133396	Tesk2	1.52	5.81E-03	DOWN	1.72	1.28E-02	DOWN
NM_181474	Tfb1m	1.34	2.60E-02	DOWN	1.87	6.10E-03	DOWN
NM_001134714	Tfcp2	1.64	1.89E-02	DOWN	1.67	4.38E-02	DOWN
NM_001012144	Tfg	1.46	6.24E-03	DOWN	1.53	3.10E-02	DOWN
NM_017200	Tfpi	1.39	3.14E-02	DOWN	1.42	4.94E-02	DOWN
NM_001009658	Thnsl2	1.80	1.59E-04	DOWN	1.94	7.17E-03	DOWN
NM_001106059	Thoc3	1.31	4.55E-02	DOWN	1.62	1.45E-02	DOWN
NM_001007682	Thtpa	1.38	5.37E-03	DOWN	1.46	1.92E-02	DOWN
NM_001012108	Thumpd2	1.91	1.19E-02	DOWN	1.59	4.26E-02	DOWN
NM_001108890	Ticam2	1.41	2.05E-03	DOWN	1.45	2.45E-02	DOWN
NM_032618	Timm22	1.33	1.34E-02	DOWN	1.67	8.21E-03	DOWN
NM_019352	Timm23	1.32	3.92E-02	DOWN	1.65	9.52E-03	DOWN
NM_017267	Timm44	1.31	1.17E-02	DOWN	1.54	2.80E-02	DOWN
NM_001006962	Tinf2	1.45	3.15E-02	DOWN	2.50	2.71E-04	DOWN
NM_001146035	Tlr10	1.18	4.40E-02	DOWN	1.18	1.49E-02	DOWN
NM_001017444	Tm2d2	1.37	1.38E-02	DOWN	1.92	1.54E-03	DOWN
NM_001127654	Tm6sf2	1.42	2.87E-02	DOWN	2.24	2.31E-02	DOWN
NM_001169102	Tma16	1.32	2.78E-02	DOWN	1.47	3.97E-02	DOWN
NM_001126048	Tma7	1.43	2.99E-02	DOWN	1.82	5.94E-03	DOWN
NM_001009631	Tmco1	1.30	2.18E-02	DOWN	2.26	7.49E-03	DOWN
NM_053467	Tmed10	1.26	2.80E-02	DOWN	1.52	4.49E-02	DOWN

NM_031722	Tmed2	1.23	1.96E-02	DOWN	1.61	1.43E-02	DOWN
NM_001009703	Tmed9	1.33	2.66E-02	DOWN	1.61	4.00E-02	DOWN
NM_001078647	Tmem134	1.32	1.32E-02	DOWN	1.39	3.30E-02	DOWN
NM_001010961	Tmem17	1.33	1.57E-02	DOWN	1.52	1.80E-02	DOWN
NM_001109572	Tmem179b	1.36	2.44E-02	DOWN	1.92	9.08E-03	DOWN
NM_199098	Tmem19	1.34	8.06E-03	DOWN	2.20	1.30E-02	DOWN
NM_001107819	Tmem203	1.30	4.34E-02	DOWN	1.71	9.48E-03	DOWN
NM_001106179	Tmem208	1.31	3.41E-02	DOWN	2.05	1.62E-02	DOWN
NM_001113780	Tmem222	1.51	2.53E-03	DOWN	1.49	2.36E-02	DOWN
NM_001108220	Tmem237	1.50	8.02E-04	DOWN	1.26	4.04E-02	DOWN
NM_001109528	Tmem25	1.87	1.09E-02	DOWN	1.80	1.91E-02	DOWN
NM_001170549	Tmem256	1.40	4.20E-02	DOWN	1.78	6.53E-03	DOWN
NM_001014192	Tmem39b	1.39	4.68E-03	DOWN	1.62	1.49E-02	DOWN
NM_001012358	Tmem41b	1.23	4.29E-02	DOWN	1.84	8.44E-03	DOWN
NM_001127525	Tmem50a	1.27	4.73E-02	DOWN	1.82	9.40E-03	DOWN
NM_001109273	Tmem51	1.66	1.46E-02	DOWN	1.82	1.52E-02	DOWN
NM_001191610	Tmem60	1.46	1.78E-03	DOWN	1.96	9.16E-04	DOWN
NM_001109258	Tmem70	1.47	1.15E-03	DOWN	1.64	3.56E-03	DOWN
NM_001017490	Tmem81	1.25	3.66E-02	DOWN	1.39	2.00E-02	DOWN
NM_001109604	Tmem86b	1.33	4.19E-02	DOWN	2.73	2.60E-02	DOWN
NM_001109299	Tmie	1.39	4.69E-03	DOWN	1.44	2.62E-02	DOWN
NM_012887	Tmpo	1.25	3.06E-02	DOWN	1.57	1.78E-02	DOWN
NM_001135858	Tmtc3	1.19	3.09E-02	DOWN	1.81	2.12E-04	DOWN
NM_198781	Tmub1	1.94	2.77E-02	DOWN	2.63	2.55E-02	DOWN
NM_001031651	Tmub2	1.38	8.58E-03	DOWN	1.68	2.43E-02	DOWN
NM_001137633	Tnfaip2	2.99	4.11E-03	DOWN	2.14	4.44E-02	DOWN
NM_001024349	Tnfrsf18	1.11	8.32E-03	DOWN	1.19	1.87E-04	DOWN
NM_133317	Tob1	1.25	1.07E-02	DOWN	1.67	1.81E-02	DOWN
NM_001002798	Top1mt	1.43	3.20E-03	DOWN	1.78	1.41E-02	DOWN
NM_001100858	Top2b	1.26	1.86E-02	DOWN	1.68	6.36E-03	DOWN
NM_139332	Tpcn1	1.40	4.84E-03	DOWN	1.71	1.19E-02	DOWN
NM_001106421	Tpd52	1.30	1.78E-02	DOWN	1.42	4.44E-02	DOWN
NM_001109621	Tpgs1	1.35	1.99E-02	DOWN	1.78	2.00E-02	DOWN
NM_001014147	Tpgs2	1.31	2.20E-02	DOWN	1.88	4.29E-04	DOWN
NM_001134994	Tpk1	1.31	1.27E-02	DOWN	1.76	2.50E-02	DOWN
NM_053534	Tpra1	1.44	5.94E-03	DOWN	1.73	1.67E-02	DOWN
NM_001024309	Tprn	1.77	2.39E-03	DOWN	1.78	1.84E-02	DOWN
NM_001012204	Traf3ip1	1.28	1.94E-02	DOWN	1.56	2.28E-05	DOWN
NM_001044248	Traf3ip2	1.35	1.51E-02	DOWN	1.87	2.33E-03	DOWN
NM_001039001	Trap1	1.33	1.53E-02	DOWN	1.66	2.62E-02	DOWN
NM_001039378	Trappc1	1.43	6.01E-03	DOWN	1.75	1.79E-02	DOWN

NM_001169115	Trappc11	1.25	3.58E-02	DOWN	1.61	1.13E-02	DOWN
NM_001106193	Trappc2l	1.39	3.07E-02	DOWN	1.40	2.22E-02	DOWN
NM_001008376	Trappc3	1.29	4.06E-02	DOWN	1.57	1.36E-02	DOWN
NM_001108276	Trim11	1.32	4.67E-02	DOWN	1.76	1.13E-02	DOWN
NM_001108552	Trim2	1.52	2.50E-02	DOWN	2.71	6.50E-04	DOWN
NM_001100637	Trim23	1.28	2.41E-02	DOWN	1.39	4.70E-02	DOWN
NM_001011665	Trim26	1.36	2.91E-03	DOWN	1.98	1.26E-02	DOWN
NM_001276491	Trim34	1.27	4.12E-02	DOWN	1.85	3.01E-02	DOWN
NM_001142944	Trim43a	1.28	4.64E-02	UP	1.20	4.98E-02	DOWN
NM_001014023	Trim5	1.46	7.86E-04	DOWN	1.97	6.97E-03	DOWN
NM_001130061	Trmt44	1.44	3.59E-02	DOWN	2.23	2.22E-03	DOWN
NM_001136229	Trpm4	1.45	2.94E-02	DOWN	2.38	1.20E-03	UP
NM_053705	Trpm7	1.34	2.39E-02	DOWN	1.97	5.48E-03	DOWN
NM_017207	Trpv2	1.09	1.95E-02	DOWN	1.47	1.89E-02	UP
NM_053686	Trpv6	1.27	1.09E-02	DOWN	1.54	5.32E-05	DOWN
NM_001105907	Trrap	1.36	9.54E-03	DOWN	1.45	1.33E-02	DOWN
NM_001009965	Tsku	2.24	1.92E-02	UP	3.12	1.34E-02	DOWN
NM_001107750	Tspan18	1.38	4.90E-02	DOWN	1.78	8.04E-04	DOWN
NM_001008378	Tspan31	1.42	2.27E-02	DOWN	2.83	2.56E-03	DOWN
NM_001013070	Tspan4	1.51	4.77E-02	UP	1.87	4.05E-02	DOWN
NM_001191618	Tspyl2	1.49	4.04E-03	DOWN	1.35	4.95E-02	DOWN
NM_001115027	Tsr2	1.33	3.56E-02	DOWN	1.48	2.28E-02	DOWN
NM_001012192	Tssc1	1.41	1.24E-02	DOWN	1.82	4.70E-03	DOWN
NM_001013194	Tssc4	1.39	2.43E-02	DOWN	1.55	3.31E-02	DOWN
NM_012808	Tst	1.33	1.63E-02	DOWN	2.29	3.12E-02	DOWN
NM_001107766	Ttbk2	1.23	3.94E-02	DOWN	1.22	4.11E-02	DOWN
NM_001025681	Ttc23	1.55	1.29E-02	DOWN	1.77	2.61E-02	DOWN
NM_001106706	Ttc27	1.42	7.04E-03	DOWN	1.60	1.17E-02	DOWN
NM_001127607	Ttc30b	1.42	7.65E-03	DOWN	1.56	1.22E-02	DOWN
NM_001005546	Ttc36	1.59	3.35E-02	DOWN	2.75	4.04E-02	DOWN
NM_001007693	Ttc9c	1.26	4.09E-02	DOWN	1.43	3.15E-02	DOWN
NM_001106295	Tufm	1.45	2.73E-03	DOWN	1.61	6.83E-03	DOWN
NM_001109297	Tusc2	1.23	4.06E-02	DOWN	1.62	7.42E-03	DOWN
NM_053331	Txn2	1.32	2.95E-02	DOWN	1.71	1.05E-02	DOWN
NM_001024998	Txndc15	1.22	4.40E-02	DOWN	1.67	1.57E-02	DOWN
NM_172032	Txndc9	1.26	4.97E-02	DOWN	1.75	7.18E-03	DOWN
NM_001257347	Tyk2	1.43	1.69E-02	DOWN	1.88	1.56E-02	DOWN
NM_017092	Tyro3	1.55	3.58E-03	DOWN	1.35	3.14E-02	DOWN
NM_001107137	Tyw1	1.50	5.55E-03	DOWN	1.73	3.35E-02	DOWN
NM_138895	Ubb	1.32	1.44E-02	DOWN	1.72	1.07E-02	DOWN
NM_001013933	Ube2a	1.39	4.38E-02	DOWN	1.74	7.57E-03	DOWN

NM_001108371	Ube2e2	1.38	2.53E-02	DOWN	1.59	2.63E-02	DOWN
NM_001047857	Ube2e3	1.42	3.80E-03	DOWN	1.57	7.54E-03	DOWN
NM_001108847	Ube2l3	1.30	2.06E-02	DOWN	1.68	4.94E-03	DOWN
NM_053928	Ube2n	1.30	3.92E-02	DOWN	1.56	1.03E-02	DOWN
NM_001110345	Ube2v1	1.37	2.06E-02	DOWN	1.49	4.71E-02	DOWN
NM_001034829	Ubxn1	1.37	4.70E-02	DOWN	1.61	1.33E-02	DOWN
NM_001107905	Ubxn2b	1.32	3.09E-02	DOWN	1.46	4.34E-02	DOWN
NM_001012025	Ubxn4	1.37	4.30E-02	DOWN	1.68	3.07E-02	DOWN
NM_001106086	Ubxn8	1.27	1.44E-02	DOWN	1.90	3.84E-03	DOWN
NM_001012149	Uchl5	1.39	4.49E-02	DOWN	1.64	5.53E-03	DOWN
NM_001003709	Ufc1	1.31	3.64E-02	DOWN	1.85	4.30E-03	DOWN
NM_001135869	Ugt2a3	2.42	6.55E-03	DOWN	4.29	1.48E-02	DOWN
NM_173323	Ugt2b7	1.87	2.35E-02	DOWN	1.76	1.58E-02	DOWN
NM_017293	Uhmk1	1.52	1.41E-02	DOWN	1.69	1.73E-03	DOWN
NM_001013884	Uimc1	1.26	3.88E-02	DOWN	1.41	3.40E-02	DOWN
NM_017188	Unc119	1.47	9.03E-03	DOWN	1.62	1.34E-02	DOWN
NM_199407	Unc5c	1.35	3.10E-02	DOWN	1.35	1.81E-02	DOWN
NM_001107319	Unc5d	1.21	3.63E-02	DOWN	1.27	2.01E-02	DOWN
NM_031777	Usf1	1.29	3.78E-02	DOWN	1.65	1.27E-02	DOWN
NM_031139	Usf2	1.31	1.00E-02	DOWN	1.39	4.74E-02	DOWN
NM_001271206	Usp24	1.25	3.73E-02	DOWN	1.45	3.81E-02	DOWN
NM_001136470	Usp49	1.80	1.16E-03	DOWN	1.97	3.72E-03	DOWN
NM_001135923	Usp9x	1.29	3.11E-02	DOWN	1.55	1.11E-02	DOWN
NM_134380	Ust5r	1.57	2.23E-02	DOWN	3.13	1.63E-02	DOWN
NM_001134558	Uvssa	1.33	2.94E-02	DOWN	1.42	1.44E-02	DOWN
NM_213563	Vars2	1.35	6.48E-03	DOWN	1.55	1.16E-02	DOWN
NM_053864	Vcp	1.41	1.44E-02	DOWN	1.64	1.26E-02	DOWN
NM_001099503	Vom2r52	1.51	1.69E-02	DOWN	1.89	5.74E-03	DOWN
NM_001173451	Vps25	1.34	3.75E-02	DOWN	1.84	1.07E-02	DOWN
NM_001106809	Vps26b	1.45	1.89E-02	DOWN	1.53	4.12E-02	DOWN
NM_001130492	Vps28	1.33	1.79E-02	DOWN	1.39	4.29E-02	DOWN
NM_022961	Vps33a	1.29	2.33E-02	DOWN	1.58	8.19E-03	DOWN
NM_001106092	Vps36	1.36	1.41E-02	DOWN	1.53	1.79E-02	DOWN
NM_001105928	Vps37b	1.85	6.85E-03	UP	1.60	4.52E-02	DOWN
NM_173147	Vps54	1.30	2.78E-02	DOWN	1.71	5.54E-03	DOWN
NM_001106600	Wbp1	1.44	1.57E-02	DOWN	1.44	4.39E-02	DOWN
NM_001108332	Wbscr16	1.36	1.49E-02	DOWN	1.64	4.07E-03	DOWN
NM_001107269	Wdfy2	1.26	8.99E-03	DOWN	2.04	2.27E-03	DOWN
NM_001107255	Wdhd1	1.20	7.49E-03	DOWN	1.53	2.09E-02	DOWN
NM_001191084	Wdr24	1.49	8.74E-03	DOWN	1.50	1.86E-02	DOWN
NM_001006988	Wdr6	1.81	1.02E-02	DOWN	1.91	2.05E-02	DOWN

NM_001107525	Wdr73	1.36	3.67E-02	DOWN	1.45	4.70E-02	DOWN
NM_001105947	Wdr83os	1.32	4.67E-02	DOWN	1.52	3.48E-02	DOWN
NM_001014078	Wdr89	1.46	9.72E-03	DOWN	1.70	5.14E-03	DOWN
NM_001107908	Wdtc1	1.44	5.86E-03	DOWN	1.68	1.16E-02	DOWN
NM_001129776	Wfikkn1	1.30	1.49E-02	DOWN	1.46	5.01E-03	DOWN
NM_001014262	Wrap73	1.39	4.78E-03	DOWN	1.63	4.81E-03	DOWN
NM_001106184	Wwp2	1.44	4.43E-03	DOWN	1.45	4.14E-02	DOWN
NM_001012099	Xkr8	1.40	3.77E-02	DOWN	1.62	3.17E-02	DOWN
NM_001107874	Xpc	1.29	2.38E-02	DOWN	1.59	1.52E-02	DOWN
NM_131913	Xpnpep1	1.28	4.78E-02	DOWN	1.63	1.31E-02	DOWN
NM_001011935	Xpo6	1.29	2.75E-02	DOWN	1.52	1.55E-02	DOWN
NM_001108386	Xpo7	1.27	4.91E-02	DOWN	1.53	1.98E-02	DOWN
NM_177419	Xrcc5	1.47	2.99E-03	DOWN	1.31	1.64E-02	DOWN
NM_022296	Xylt2	1.66	1.81E-03	DOWN	1.95	1.64E-02	DOWN
NM_001108529	Ybey	1.26	1.98E-02	DOWN	1.63	1.18E-02	DOWN
NM_001109057	Yeats2	1.38	2.28E-02	DOWN	1.63	1.36E-02	DOWN
NM_001127527	Yeats4	1.55	9.37E-03	DOWN	1.65	1.71E-02	DOWN
NM_172017	Yif1a	1.48	9.54E-03	DOWN	1.83	1.17E-02	DOWN
NM_001014208	Yipf2	1.49	1.03E-02	DOWN	1.60	3.91E-02	DOWN
NM_001007801	Yipf3	1.31	2.25E-02	DOWN	1.62	2.01E-02	DOWN
NM_001009712	Yipf4	1.22	1.22E-02	DOWN	1.79	3.38E-03	DOWN
NM_001106129	Zadh2	1.43	9.30E-03	DOWN	1.88	1.65E-02	DOWN
NM_001009172	Zbtb22	1.43	1.90E-02	DOWN	1.50	1.64E-02	DOWN
NM_001106657	Zbtb5	1.41	9.46E-03	DOWN	1.48	3.76E-02	DOWN
NM_213564	Zbtb9	1.34	1.43E-02	DOWN	1.45	1.29E-02	DOWN
NM_001108146	Zc3h12c	1.21	1.81E-02	DOWN	1.28	5.40E-03	DOWN
NM_001126374	Zc4h2	1.41	1.59E-02	DOWN	1.73	5.23E-04	DOWN
NM_001108360	Zcchc4	1.48	2.83E-03	DOWN	1.53	2.29E-02	DOWN
NM_001013239	Zdhhc12	1.31	2.52E-02	DOWN	1.94	6.00E-03	DOWN
NM_001013123	Zdhhc4	1.35	3.46E-02	DOWN	1.82	4.91E-03	DOWN
NM_001100707	Zer1	1.43	1.06E-03	DOWN	1.51	1.54E-02	DOWN
NM_173332	Zfand4	1.31	2.22E-02	DOWN	1.50	2.55E-02	DOWN
NM_001135018	Zfp266	1.21	2.27E-02	DOWN	1.47	1.82E-02	DOWN
NM_001107736	Zfp385b	1.54	3.93E-02	DOWN	2.53	5.16E-03	DOWN
NM_019620	Zfp386	1.41	1.39E-02	DOWN	2.07	2.13E-03	DOWN
NM_001079943	Zfp426	1.62	7.39E-04	DOWN	1.37	9.05E-03	DOWN
NM_001169143	Zfp444	1.63	3.14E-04	DOWN	1.68	3.53E-03	DOWN
NM_001191802	Zfp445	1.28	4.25E-02	DOWN	1.63	1.53E-02	DOWN
NM_001107809	Zfp512b	1.40	6.99E-03	DOWN	1.99	3.11E-04	DOWN
NM_001126272	Zfp692	1.33	3.86E-02	DOWN	1.51	3.89E-02	DOWN
NM_001108063	Zfp763	1.81	2.01E-04	DOWN	1.42	8.63E-03	DOWN

NM_001107472	Zfp772	1.30	2.67E-02	DOWN	1.53	1.09E-02	DOWN
NM_001134544	Zfp865	1.40	1.78E-02	DOWN	1.69	2.95E-03	DOWN
NM_001134600	Zfp879	1.35	2.64E-02	DOWN	1.45	4.34E-02	DOWN
NM_001142758	Zfp958	1.47	1.35E-02	DOWN	2.05	5.52E-03	DOWN
NM_001286936	Zfpl1	1.42	1.45E-02	DOWN	1.45	4.75E-02	DOWN
NM_001135582	Zmat2	1.48	6.81E-03	DOWN	1.76	9.42E-03	DOWN
NM_001107983	Zmym1	1.41	3.75E-02	DOWN	1.62	1.35E-02	DOWN
NM_001040155	Zmym3	1.43	1.18E-02	DOWN	1.61	7.08E-03	DOWN
NM_001004284	Zmynd10	1.39	4.15E-02	DOWN	1.66	2.73E-02	DOWN
NM_001271350	Znhit1	1.47	1.63E-02	DOWN	1.54	2.91E-02	DOWN
NM_001108628	Znrf2	1.32	2.29E-02	DOWN	2.01	4.07E-02	DOWN
NM_001012021	Zscan21	1.30	2.87E-02	DOWN	1.56	4.37E-03	DOWN
NM_001107163	Zswim4	1.43	1.06E-02	DOWN	1.47	3.20E-02	DOWN

Table S2. Male DEG in common from the comparisons MO-110PND vs C-110PND and C-650PND vs C-110PND in males. Log2Fold change regulation and their statistical significance are shown by P-value.

		**MO110 vs C110**			**650 vs c110**		
**Gene ID**	**Gene**	Fold Regulation	P-value (t-test)	Direction	Fold Regulation	P-value (t-test)	Direction
NM_001111056	Adarb1	1.9	2.63E-02	UP	1.64	1.93E-02	UP
NM_001107322	Ank1	1.1	2.04E-02	DOWN	1.11	1.13E-02	DOWN
NM_031780	Apba2	2.7	9.08E-04	UP	1.96	1.96E-02	UP
NM_001077201	Caln1	1.1	4.68E-03	DOWN	1.85	8.72E-04	UP
NM_053874	Cap2	1.1	2.70E-02	DOWN	1.40	2.50E-02	UP
NM_001110808	Capn12	1.4	4.82E-02	UP	1.43	1.23E-02	UP
NM_012552	Cela1	2.9	3.14E-03	DOWN	2.48	1.77E-03	DOWN
NM_001109353	Clec12b	1.2	5.22E-03	UP	1.19	2.38E-02	UP
NR_037674	Cox6a2	1.1	3.62E-02	UP	1.05	4.95E-02	UP
NM_012536	Ctrb1	1.1	4.34E-02	DOWN	1.13	2.66E-02	DOWN
NM_001100908	Fam129c	1.9	2.81E-02	UP	2.00	1.91E-02	UP
NM_001005888	Galc	1.4	2.98E-02	UP	1.93	1.38E-02	UP
NM_001145842	Grn	1.5	4.34E-02	UP	1.63	2.08E-02	UP
NM_001005900	Hcst	1.3	2.18E-02	UP	1.53	2.15E-02	UP
NM_013122	Igfbp2	2.7	3.66E-02	DOWN	2.84	9.03E-03	UP
NM_181634	Lppr3	1.3	3.28E-02	DOWN	5.71	1.33E-03	UP
NM_001009497	Ly49si1	1.1	2.46E-02	UP	1.24	1.77E-02	UP
NM_001106338	Ms4a7	1.2	4.88E-02	UP	1.36	7.18E-03	UP

NM_173096	Mx1	1.5	4.56E-02	UP	1.17	2.41E-02	UP
NM_001270867	Nnat	1.0	3.13E-02	UP	1.03	3.84E-02	UP
NM_001271129	Nrg1	1.2	1.55E-02	DOWN	1.40	4.57E-02	UP
NM_024128	Nsg1	1.2	4.21E-02	DOWN	1.81	7.48E-03	UP
NM_001033959	Obp3	1.6	4.22E-03	DOWN	1.56	4.18E-03	DOWN
NM_001106024	Pde6b	1.1	2.06E-02	UP	1.10	3.32E-02	UP
NM_001108522	Pde6c	1.5	4.50E-04	UP	1.73	1.86E-03	UP
NM_001145366	Pparg	1.3	1.03E-02	UP	1.39	4.18E-02	UP
NM_198773	Ppm1e	1.1	2.16E-02	DOWN	1.09	2.99E-10	DOWN
NM_001109328	Rab7b	1.6	2.80E-02	UP	1.57	3.32E-02	UP
NM_053721	Rasgrf2	1.2	2.30E-02	UP	1.17	3.27E-03	UP
NR_027235	RGD1559747	1.3	4.09E-02	UP	1.26	1.01E-02	UP
NM_001127567	RGD1561648	1.2	3.59E-02	UP	1.40	2.45E-02	UP
NM_001172116	Samd3	1.3	2.21E-02	UP	2.28	7.32E-03	UP
NM_001108964	Spo11	1.1	3.85E-02	UP	1.06	1.04E-02	UP
NM_001271235	Tnfrsf11a	1.7	1.66E-02	UP	1.62	9.75E-03	UP
NM_001109112	Tnfsf13b	1.2	4.64E-02	UP	1.40	1.68E-03	UP
NM_001170559	Vcan	1.2	1.57E-02	UP	1.26	4.40E-02	UP
NM_001013863	Ydjc	1.1	1.66E-02	UP	1.21	6.61E-03	UP

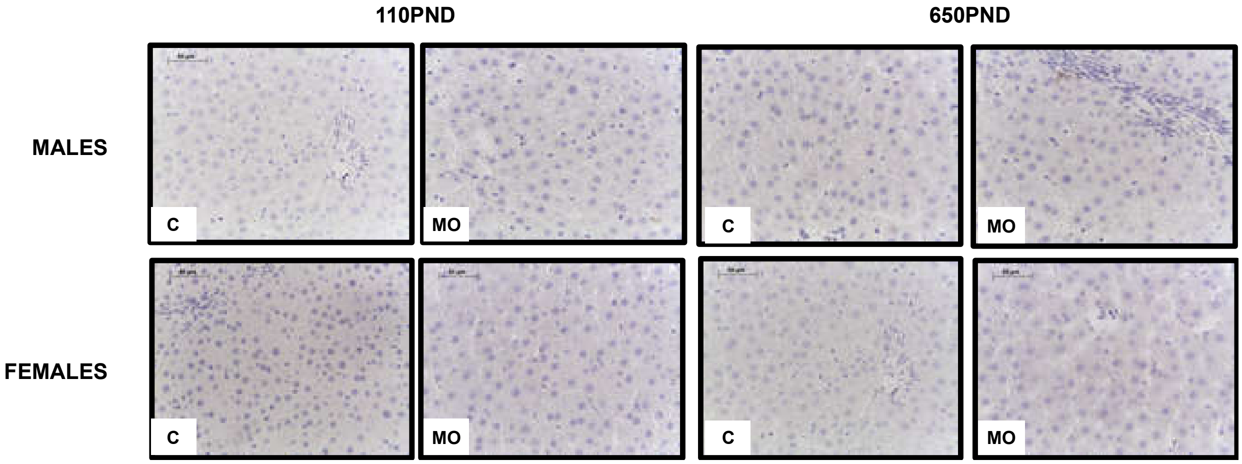

Figure S1. Representative IHC micrograph of negative controls (40x).
